# The Invasive Species *Reynoutria japonica* Houtt. as a Promising Natural Agent for Cardiovascular and Digestive System Illness

**DOI:** 10.3389/fphar.2022.863707

**Published:** 2022-06-13

**Authors:** Shaoyang Liu, Ruiyuan Zhang, Xing Zhang, Shun Zhu, Siyu Liu, Jue Yang, Zhiping Li, Tianhui Gao, Fang Liu, Huiling Hu

**Affiliations:** ^1^ State Key Laboratory of Southwestern Chinese Medicine Resources, College of Pharmacy, Chengdu University of Traditional Chinese Medicine, Chengdu, China; ^2^ Sichuan Quantaitang Chinese Herbal Slices Co, Ltd., Chengdu, China

**Keywords:** *Reynoutria japonica* Houtt., botany and ethnopharmacology, phytochemistry, pharmacological activity, quality control

## Abstract

Polygoni Cuspidati Rhizoma et Radix, the dry roots and stems of *Reynoutria japonica* Houtt (called Huzhang, HZ in Chinese), is a traditional and popular chinese medicinal herb for thousands of years. As a widely used ethnomedicine in Asia including China, Japan, and Korea, HZ can invigorate the blood, cool heat, and resolve toxicity, which is commonly used in the treatment of favus, jaundice, scald, and constipation. However, HZ is now considered an invasive plant in the United States and many European countries. Therefore, in order to take advantage of HZ and solve the problem of biological invasion, scholars around the world have carried out abundant research studies on HZ. Until now, about 110 compounds have been isolated and identified from HZ, in which anthraquinones, stilbenes, and flavonoids would be the main bioactive ingredients for its pharmacological properties, such as microcirculation improvement, myocardial protective effects, endocrine regulation, anti-atherosclerotic activity, anti-oxidant activity, anti-tumor activity, anti-viral activity, and treatment of skin inflammation, burns, and scalds. HZ has a variety of active ingredients and broad pharmacological activities. It is widely used in health products, cosmetics, and even animal husbandry feed and has no obvious toxicity. Efforts should be made to develop more products such as effective drugs, health care products, cosmetics, and agricultural and animal husbandry products to benefit mankind.

## Graphical Abstract

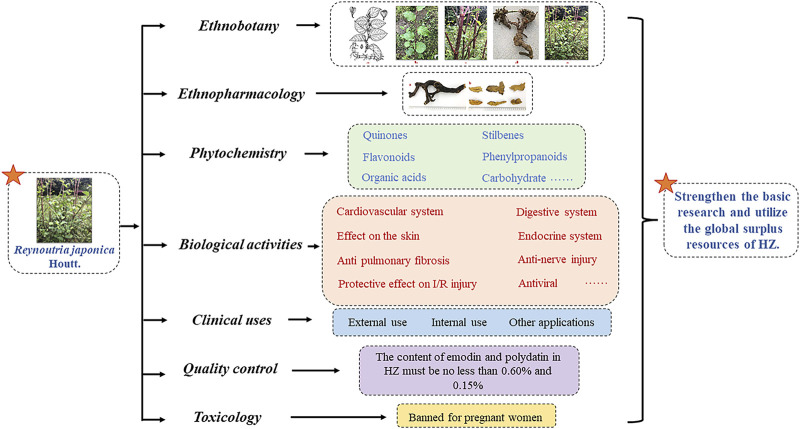

## 1 Introduction

*Reynoutria japonica* Houtt. (called Huzhang, HZ in Chinese), also known as *Polygonum cuspidatum* Sieb. et Zucc. and *Fallopia japonica* (Houtt.) Ronse Decr, is the main source of a traditional and popular Chinese medicinal herb named Polygoni Cuspidati Rhizoma et Radix (*Reynoutria japonica* Houtt. 2021). The dry roots and stems of HZ can be used for treatment of favus, jaundice, scald, constipation, and so on*.* However, HZ is now considered an invasive plant in the United States and many European countries and is one of the plants banned by law from planting in the wild in the United Kingdom due to its aggressive growth, allelopathic effects, and extremely strong abiotic stress tolerance (Weston et al., 2005) (Dawsonl et al., 1999). This plant, which is native to Japan, was introduced to Britain as an ornamental in 1825 and soon became an epidemic (Guo 2015). It is considered one of the most ecologically and economically damaging invasive non-native plants in the United Kingdom, where it is widespread in a variety of habitats (Engler et al., 2011) (Gozlan et al., 2013). The main hazards of HZ to invasive sites include the formation of a single dominant population to replace native plants, resulting in the loss of habitat for many native plants (Wilson et al., 2017). This could threaten local biodiversity, invade grasslands and roads and damage the local economy. HZ is listed as one of the 100 most destructive invasive species in the world by the International Union for Conservation of Nature. Currently, it is estimated that the annual damage caused by invasive weeds to America is up to $35 billion (Weston et al., 2005). In 2010, HZ was estimated to have cost the UK economy of £165 million (Robinson et al., 2016).

HZ has been introduced into China for about 2,000 years and was first recorded in the “*Miscellaneous Records of Famous Physicians*” in the late Han Dynasty (B.C. 202-A.D. 220) (Cui 1998). Traditionally, it is believed that HZ can invigorate the blood, cool heat, and resolve toxicity by means of using alone or in combination with other herbs. HZ has been recorded in the Chinese Pharmacopoeia (ChP.) since 1977. After washing and slicing, the dry roots and stems could be used as medicine. In China and Japan, the roots of HZ have been used in treatment of inflammation, infection, jaundice, skin burns, and hyperlipemia diseases (Peng et al., 2013). Statistically, 140 Chinese patent medicines (CPMs) containing HZ have been developed on the basis of the data of National Medical Products Administration (NMPA, http://www.nmpa.gov.cn/). Clinically, the processed product-wine-fried HZ is also very effective. The herb is mixed with yellow rice wine, left covered briefly as it is absorbed, and then dry-fried until slightly scorched. This method of preparation strengthens its ability to invigorate the blood and soothe the sinews, while reducing its slight tendency to irritate the digestion (Bensky et al., 2004).

Modern pharmacological studies have confirmed that HZ possesses wide pharmacological actions such as effects on endocrine and cardiovascular systems, anti-tumor, anti-viral, anti-oxidation, and anti-pulmonary fibrosis. Due to its extensive pharmacological effects and universal folk use, the research studies on HZ chemical components get more and more attention. Until now, more than 100 compounds have been isolated and identified from HZ, including quinones, aromatic hydrocarbons, flavonoids, and other less abundant ingredients such as phenylpropanoids and organic acids. Among them, anthraquinones and stilbenes have been the most extensively studied and possess the notable bioactivity. In 2020 Edition ChP., emodin (2) and polydatin (26) are now used as the official markers to monitor the quality of the stems and roots. Herein, in order to make better use of the global surplus resource of HZ, we have a detailed description on HZ. An extensive review of the literature was conducted, and electronic databases including the Web of Science, ScienceDirect, PubMed, Google Scholar, Baidu Scholar, and CNKI by using the keywords “*Reynoutria japonica* Houtt.“, “*Polygonum cuspidatum* Sieb. et Zucc.”, “Huzhang”, “*Fallopia japonica* (Houtt.) Ronse Decr”, “emodin”, “polydatin”, “quercitrin”, “resveratrol”, “quercetin”, and their combinations were used. A total of 286 studies were identified through electronic databases from their inception up to December 2021. In addition, information was collected from relevant textbooks, reviews, and documents (e.g., 2020 Edition ChP., Chinese herbal classic books and PhD and MSc theses, *etc.*), which compiles a variety of literature studies and website information to provide comprehensive insights into the ethnopharmacology, phytochemistry, pharmacological activity, clinical use, quality control, and toxicology of HZ in this review for further in-depth development and applications.

## 2 Ethnobotany

**FIGURE 1:**
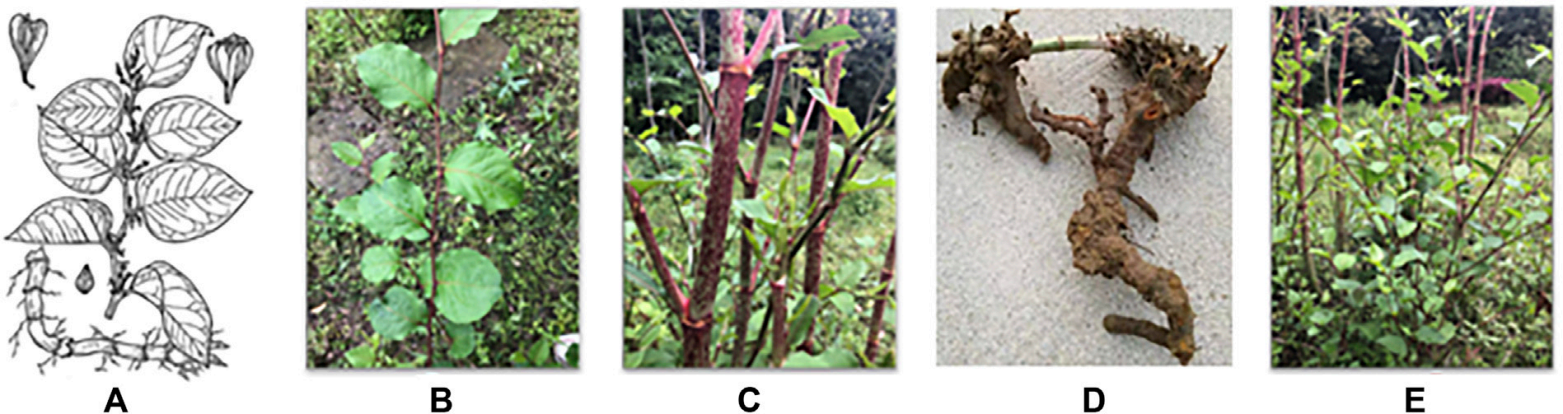
Schematic diagram of HZ herbs. **(A)** Hand-painted whole-plant image of HZ, **(B)** HZ leaves, **(C)** HZ stems, **(D)** HZ roots and rhizomes, and **(E)** aerial view of HZ.

HZ mainly grows in valleys, ditches, roadsides, wetlands, and other humid habitats, distributed between 1 m above the sea level (Wang 2008). It belongs to the Polygonaceae family of eudicots, which includes many other key medicinal plants, such as *Rheum palmatum* (Chinese rhubarb), *Polygonum multiflorum*, and *Fagopyrum cymosum* (tall buckwheat), as well as the pseudocereal crop *Fagopyrum tataricum* (Tartary buckwheat). HZ is a perennial herb with thickened rhizomes. Stems are numerous, erect, 1–2 m tall, stout, branched above, striate, papillate, and often with red or purple spots. Leaves are often deciduous with petioles of 1–2 cm, papillate with the leaf blade ovate or broadly elliptic, 5−12 × 4–9 cm, subleathery, with both surfaces glabrous, papillate along veins, with the base broadly cuneate, rounded, or truncate and the margin entire, apex acute, or shortly acuminate, not ciliate. Inflorescence is axillary, paniculate, 3–8 cm; bracts are funnel-shaped, 1–2 mm, and oblique, with each 2–4-flowered. Pedicels are 3–4 mm, slender, and articulate below middle. Perianth is white or greenish and 5-parted. Male flowers: eight stamens, longer than the perianth. Female flowers: three outer tepals accrescent and winged on the abaxial surface; three styles; stigmas fimbriate. Achenes are included in the persistent perianth, black-brown, shiny, ovoid-ellipsoid, 4–5 mm. Flowering is in June–September, and fruiting is in July–October (Figure 1). (Reynoutria, 1998).

This plant is native in eastern Asia such as China, Japan, and Korea (Li et al., 2019a). It is widely cultivated as the essential medicinal plant in many provinces of China including Anhui, Fujian, Gansu, Guangdong, Guangxi, and so on In contrast to its medicinal uses, HZ is regarded as an invasive plant in Europe and North American. HZ grows and reproduces very quickly and relies on asexual reproduction mainly. HZ has extremely strong vitality, and its underground rhizome system is very developed. A small section of rhizome can quickly grow into a complete plant. Because of its strong penetrating ability, it can drill out from the cement slabs or brick cracks and prop up the cracks in the building due to its strong roots. Therefore, it becomes the “killer” of roads, bridges, building foundations, flood control dikes, sewers, and so on (Fanny et al., 2016). However, in 2017, the rhizome of HZ was included in the European Pharmacopoeia (Nawrot-Hadzik et al., 2018). This means that people gradually realized the beneficial side of HZ to humans. Therefore, we should actively research and continuously develop new uses of HZ to turn waste into treasure.

## 3 Ethnopharmacology

**FIGURE 2:**
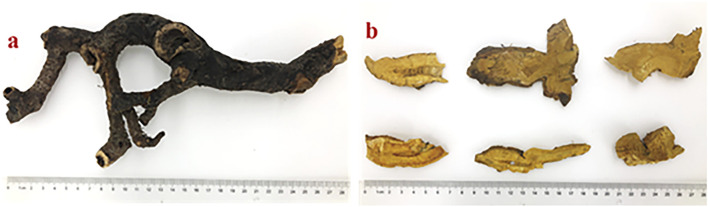
Schematic diagram of medicine material crude slices of HZ. **(A)** Whole medicinal parts and **(B)** sliced medicinal parts.

In China, the frequently used prepared herbal medicine in small pieces ready for decoction of HZ clinically refers to its dried rhizomes and roots (underground parts). HZ is mostly in cylindrical short sections or irregular thick slices, 1–7 cm long, 0.5–2.5 cm in diameter from the appearance, And externally brown, showing longitudinal wrinkles and rootlet scars. In the transversely cut surface, the bark is relatively thin, the wood is broad and brownish-yellow with radial rays, and the bark can be easily separated from the wood. The pith in a rhizome is septated or hollowed (Figure 2). The texture is hard. The odor is slight; the taste is slightly bitter and astringent (NCoC 2020). TCM holds that HZ is bitter in taste and cool in nature and acts on the liver, gallbladder, and lung channels. It invigorates the blood, eliminates wind and humidity, transforms phlegm, cools heat, and resolves toxicity (Bensky et al., 2004). It applies to cure amenorrhea and wind-damp painful obstruction (Zhou et al., 2020c), traumatic injury (Zhang et al., 1997), and cough due to lung heat. It is also used for burns, carbuncles (Yang 2010), other skin infections, and snakebite (Wang ZQ. et al., 2010). Usually, the fresh ground herb is applied locally for these problems in addition to any internal consumption.

The classic method of using HZ alone to treat children’s fever and night sweats is included in ‘Xiao Er Yao Zheng Zhi Jue’, which is a famous monograph on pediatrics of traditional Chinese medicine (TCM) written in 1119 A.D. Statistically, from the Eastern Han dynasty (A.D.25–A.D.220) to Modern times (since A.D.1840), there were 77 classic prescriptions containing HZ used frequently by physicians (Table 1) (Bai et al., 2016). In addition, there are some applications of HZ in classical prescriptions of TCM in the (Table 2). This shows that in the clinical practice of TCM, HZ is often used in combination with other medicinal materials to exert better curative effects (Meng et al., 2000). For example, HZ can treat stones in the biliary or urinary tracts paired with Lysimachiae Herba (called Jin Qian Cao, JQC in Chinese). Dan Dao Pai Shi Decoction IV, included in “Xin Ji Fu Zheng Xue”, which was published in 1961, can cure cholelithiasis because of containing these two herbs. HZ can also invigorate the blood, dispel stasis, and stop pain when meeting Paeoniae Radix Rubra (Chi Shao, CS in Chinese). It is recorded in “Sheng Ji Zong Lu” (A.D. 1,117) that HZ powder, composed of HZ and CS only, can treat blood stasis caused by trauma. Besides, HZ combined with Angelicae Sinensis Radix (Dang Gui, DG in Chinese) or Cinnamomi Ramulus (Gui Zhi, GZ in Chinese) is effective for patients with gouty. According to “Tai Ping Sheng Hui Fang” (A.D.992), in the collection of HZ powder which contains HZ, GZ, and DG mainly, the channels can be unblocked to cure wind-damp painful obstruction. Modern pharmacological studies have proved the antigout effects of the HZ-GZ herb pair in acute gouty arthritis rat models (wang). Zhou also found that HZ Tongfeng granules which mainly contain HZ and DG could partially attenuate the inflammation induced by monosodium urate (MSU) crystals (Zhou et al., 2020c).

**TABLE 1:** Utilization of various functions of HZ in different historical periods.

Dynasty	Resolves Dampness and Treats Jaundice	Transforms Phlegm and Stops Coughs	Clears Heat and Resolves Toxicity	Invigorates the Blood and Unblocks the Channels	Summation
Tang (A.D. 618–907)	—	—	—	3	3
Song (A.D. 960–1,279)	8	—	6	31	45
Yuan (A.D.1272–1,368)	—	—	—	—	0
Ming (A.D. 1,368–1,644)	2	—	5	1	8
Qing (A.D. 1,636–1921)	1	—	—	4	5
Modern times (since 1840)	3	3	7	3	16
Summation	14	3	18	42	77

**TABLE 2:** Application of HZ in classical prescriptions of TCM.

Prescription Name	Main Herbs	Traditional Use	Dosage of HZ (g)	References	Dynasty
Dan Dao Pai Shi	Aurantii Fructus	Curing cholelithiasis	25	*Xin Ji Fu Zheng Xue*	Modern times (A.D. 1961)
Decoction IV	Aucklandiae Radix				
	Scutellariae Radix				
	Polygoni Cuspidati Rhizoma et Radix				
Li Dan Pai Shi	Rhei Radix et Rhizoma	Regulating liver Qi and helping discharge gallstones	15	*Jiang Chun Hua Fang*	Modern times (A.D. 1908–1992)
Decoction	Aurantii Fructus Immaturus				
	Curcumae Radix				
	Lysimachiae Herba				
	Polygoni Cuspidati Rhizoma et Radix				
Hu Zhang	Paeoniae Radix Rubra	Treating blood stasis caused by trauma	117	*Sheng Ji Zong Lu*	Song (A.D. 1,117)
Powder	Polygoni Cuspidati Rhizoma et Radix				
Hu Zhang	Akebiae Caulis	Invigorating the blood and stopping pain due to abdominal blood stasis	78	*Sheng Ji Zong Lu*	Song (A.D. 1,117)
Decoction	Achyranthis Bidentatae Radix				
	Imperatae Rhizoma				
	Persicae Semen				
	Polygoni Cuspidati Rhizoma et Radix				
Hu Zhang	Angelicae Sinensis Radix	Curing gout	45	*Tai Ping Sheng Hui Fang*	Song (A.D. 992)
Powder	Cinnamomi Ramulus				
	Paeoniae Radix Rubra				
	Aurantii Fructus Immaturus				
	Polygoni Cuspidati Rhizoma et Radix				
Hua Du	Armeniacae Semen Amarum	Clearing heat and resolving toxicity	1.5	*Pu Ji Fang*	Ming (A.D. 1,390)
Decoctoin	Moutan Cortex				
	Scutellariae Radix				
	Ephedrae Herba				
	Polygoni Cuspidati Rhizoma et Radix				
Fei Nong Yang He Wan	Scutellariae Barbatae Herba	Stopping coughs due to lung heat and transforming phlegm	12	*Gu Jin Ming Fang*	Modern times (A.D. 2001)
Decoction	Lonicerae Japonicae Flos				
	Houttuyniae Herba				
	Scutellariae Radix				
	Polygoni Cuspidati Rhizoma et Radix				
Hu Zhang	Polygoni Cuspidati Rhizoma et Radix	Curing burns (external use)	500	*Chinese Dermatology (Abridged Edition)*	Modern times (A.D. 1982)
Decoction					
Hu Zhang Hong Yao Zi	Polygonum cillinerve (Nakai) Ohwi	Curing allergic rhinitis and skin ulceration (external use)	500	*Qian Jia Miao Fang*	Modern times (A.D. 1982)
Ointment	Borneolum Syntheticum				
	Polygoni Cuspidati Rhizoma et Radix				

Besides its therapeutic applications, HZ has been commonly used in daily food in some Asian countries. The roots of HZ have been used to dye rice flour, and the tender stems have been used as foodstuff (Kirino et al., 2012). In China, Tujia people have the habit of eating HZ as a vegetable. After being harvested in April to May each year, it can be eaten peeled (Huang et al., 2007). Moreover, pigments extracted from HZ, natural and non-toxic, are widely used as food additives (Meng et al., 2000). Among them, the yellow pigment is widely used in the cosmetics industry owing to its bright color and rich anti-oxidant substances (Barbieri et al., 2018) (Barbieri et al., 2019). In India and southeast Asia, its dry leaves are used as a kind of tobacco (Kirino et al., 2012). Now, there was one research on easy transformation of HZ into the carbon adsorbent, which is usable for sorption of diclofenac and paracetamol (Koutnik et al., 2020).

## 4 Phytochemistry

Since the early 1950s (Peng et al., 2013), researchers have used a variety of methods of extraction, separation, characterization, and identification to study the multiplicate compositions of HZ. To date, a total of 110 compounds have been isolated and assuredly identified from HZ, predominantly containing anthraquinones, naphthoquinones, stilbenes, flavonoids, and others (Table 3). Among them, anthraquinones and stilbene components are currently considered to be the main active substances for HZ with pharmacological effects (Sun et al., 2015).

**TABLE 3:** Compounds presenting in HZ.

dsludsluClassification	No	Compound	Molecular Formula	Extraction	Parts	References	PubChem CID
**Quinones**							
*Anthraquinones*	1	Physcion	C_16_H_12_O_5_	Methanol, Ultrasonic	Root, rhizome, flower, leaf	Sun and Wang. (2015)	10,639
						Wang et al. (2019c)	
	2	Emodin	C_15_H_10_O_5_	Methanol, Ultrasonic	Root, rhizome, flower, leaf	Sun and Wang. (2015)	3,220
						Wang et al. (2019c)	
	3	Fallacinol	C_16_H_12_O_6_	Methanol, Reflux	Root, rhizome	Kimura et al. (1983b)	3,083,633
	4	Questin	C_16_H_12_O_5_	Methanol, Reflux	Root	Kimura et al. (1983b)	160,717
	5	Anthraglycoside A	C_22_H_22_O_10_	Methanol, Reflux	Root, rhizome, leaf	Kimura et al. (1983b)	—
						Wang et al. (2019c)	
	6	Anthraglycoside B	C_21_H_20_O_10_	Methanol, Ultrasonic	Root, rhizome, flower	(Kimura et al., 1983b; Sun et al., 2015a)	118,855,584
	7	Chrysophanol	C_15_H_10_O_4_	Methanol, Ultrasonic	Root, flower	Sun et al. (2015a)	10,208
	8	Citreorosein	C_15_H_10_O_6_	Methanol, Reflux	Root	Kimura et al. (1983b)	361,512
	9	Questinol	C_16_H_12_O_6_	Methanol, Reflux	Root	Kimura et al. (1983b)	147,621
	10	Rhein	C_15_H_8_O_6_	Methanol, Ultrasonic	Root, flower	Sun et al. (2015a)	10,168
	11	Emodin-8-O-(6′-O-malonyl)-glucoside	C_24_H_22_O_13_	Methanol–water (80:20, v/v), Ultrasonic	Leaf	Wang et al. (2019c)	—
						Wang, (2018)	
	12	Polyganin A	C_25_H_24_O_13_	95% Ethanol, Reflux	Root	Zhang et al. (2012a)	—
	13	Polyganin B	C_26_H_26_O_13_	95% Ethanol, Reflux	Root	Zhang et al. (2012a)	—
	14	Physcion-1-O-β-D-glucoside	C_22_H_22_O_10_	Methanol, Reflux	Root	Kimura et al. (1983b)	—
	15	Emodin-1-O-β-D-glucoside	C_22_H_22_O_9_	Methanol–water (80:20, v/v), Ultrasonic	Root, leaf	Wang et al. (2019c)	—
						Wang, (2018)	
	16	Aloe-emodin	C_15_H_10_O_5_	Methanol, Ultrasonic	Flower	Sun et al. (2015a)	10,207
	17	Phylloquinone B	C_68_H_109_O_6_	None	Leaf	Wang et al. (2019c)	—
	18	Phylloquinone C	C_39_H_54_O_3_	None	Leaf	Wang et al. (2019c)	—
	19	Rubiadin	C_15_H_10_O_4_	None	None	Lin et al. (2015)	124,062
*Naphthoquinones*	20	2-Methoxy-6-acetyl-7-methyljuglone	C_14_H_12_O_5_	Methanol, Reflux	Root	(dskkkk)	158,739
	21	Cuspidatumin A	C_14_H_12_O_4_	95% Ethanol, Reflux	Root, rhizome	Jin et al. (2009)	—
	22	7-Acetyl-2-methoxy-6-methyl-8-hydroxy-1,4-naphthoquinone	C_13_H_12_O_5_	Methanol, Reflux	Root	Kimura et al. (1983b)	—
	23	2-Methoxy-6-acetylmcthljuglone	C_14_H_12_O_4_	None	Root	Ouyang (1987)	—
	24	2-Ethoxystypandrone	C_15_H_14_O_5_	Ethyl acetate, Speed extraction	Root	Li et al. (2019b)	—
**Aromatic hydrocarbons**							
*Stilbenes*	25	Resveratrol	C_14_H_12_O_3_	60% Aqueous acetone, Ultrasonic	Root, rhizome	Xiao et al. (2002)	445,154
	26	Polydatin	C_20_H_22_O_8_	60% Aqueous acetone, Ultrasonic	Root, rhizome	Xiao et al. (2002)	5,281,718
	27	Resveratrol-4′-O-glucoside	C_20_H_22_O_8_	None	Root, rhizome	(Jayatilake et al., 1993)	131,751,049
	28	Resveratrol 4-O-D-(2′-galloyl)-glucopyranoside	C_27_H_26_O_11_	70% Aqueous methanolic, Ultrasonic	Root	(Hegde et al., 2004)	—
	29	Resveratrol 4-O-D-(6′-galloyl)-glucopyranoside	C_27_H_26_O_12_	70% Aqueous methanolic, Ultrasonic	Root	(Hegde et al., 2004)	—
	30	Sodium and potassium trans-resveratrol-3- O-β-D-glucopyranoside-6″-sulfate	C_20_H_21_O_8_SO_3_Na	Aqueous acetone, Extract	Root	Xiao et al. (2002)	—
	31	Sodium and potassium trans-resveratrol-3-O-β-D-glucopyranoside-4″-sulfate	C_20_H_21_O_8_SO_3_Na	Aqueous acetone, Extract	Root	Xiao et al. (2002)	—
	32	Sodium and potassium trans-resveratrol-3-O-β-D-glucopyranoside-2″-sulfate	C_20_H_21_O_8_SO_3_Na	Aqueous acetone, Extract	Root	Xiao et al. (2002)	—
	33	Sodium and potassium trans-resveratrol-3-O-β-D-glucopyranoside-4′-sulfate	C_20_H_21_O_8_SO_3_Na	Aqueous acetone, Extract	Root	Xiao et al. (2002)	—
	34	Sodium and potassium trans-resveratrol-3-O-β-D-glucopyranoside-5-sulfate	C_20_H_21_O_8_SO_3_Na	Aqueous acetone, Extract	Root	Xiao et al. (2002)	—
	35	Sodium and potassium cis-resveratrol-3-O-β-D-glucopyranoside-6″-sulfate	C_20_H_21_O_8_SO_3_Na	Aqueous acetone, Extract	Root	Xiao et al. (2002)	—
	36	Sodium and potassium cis-resveratrol-3-O-β-D-glucopyranoside-4″-sulfate	C_20_H_21_O_8_SO_3_Na	Aqueous acetone, Extract	Root	Xiao et al. (2002)	—
	37	Sodium and potassium cis-resveratrol-3-O-β-D-glucopyranoside-3″-sulfate	C_20_H_21_O_8_SO_3_Na	Aqueous acetone, Extract	Root	Xiao et al. (2002)	—
	38	Sodium and potassium cis-resveratrol-3-O-β-D-glucopyranoside-2″-sulfate	C_20_H_21_O_8_SO_3_Na	Aqueous acetone, Extract	Root	Xiao et al. (2002)	—
	39	Sodium and potassium cis-resveratrol-3-O-β-D-glucopyranoside-5-sulfate	C_20_H_21_O_8_SO_3_Na	Aqueous acetone, Extract	Root	Xiao et al. (2002)	—
	40	Polynapstilbene A	C_39_H_42_O_17_	None	Root, rhizome	Peng et al. (2013)	—
	41	Polynapstilbene B	C_39_H_42_O_17_	None	Root, rhizome	Peng et al. (2013)	—
**Flavonoids**							
*Flavonols*	42	Rutin	C_27_H_30_O_16_	Methanol, Reflux	Flower	Jin et al. (2009)	5,280,805
	43	Reynoutrin	C_20_H_18_O_11_	None	Leaf	Peng et al. (2013)	5,320,863
	44	Kaempferol	C_15_H_10_O_6_	Methanol, Reflux	Flower	Sun et al. (2015a)	5,280,863
	45	Hyperoside	C_21_H_20_O_12_	Methanol, Reflux	Root, flower	Peng et al. (2013)	5,281,643
						Sun et al. (2019)	
	46	Isoquercitrin	C_21_H_20_O_12_	Methanol, Reflux	Root	Peng et al. (2013)	5,280,804
						Sun et al. (2019)	
	47	Quercitrin	C_21_H_20_O_11_	Methanol, Reflux	Root	Peng et al. (2013)	5,280,459
	48	Quercetin	C_15_H_10_O_7_	Methanol, Ultrasonic	Root, rhizome, flower	Sun et al. (2019)	5,280,343
	49	Querectin-3-O-arabinoside	C_20_H_18_O_10_	Methanol, Reflux	Root	Peng et al. (2013)	12,309,865
						Sun et al. (2019)	
	50	Querectin-3-O-α-L-arabinoside	C_20_H_18_O_10_	None	Leaf	Wang et al. (2019c)	—
	51	Quercetin-3-xyloside	C_20_H_18_O_11_	None	Leaf	Zhang (1999)	5,321,278
	52	Polyflavanostilbene A	C_42_H_38_O_19_	None	Plant	Li et al. (2013)	72,195,698
*Flavones*	53	Apigenin	C_15_H_10_O_5_	Water, Ultrasonic	Flower, root	Sun and wang. (2015)	5,280,443
	54	Luteolin	C_15_H_10_O_6_	None	Root	Peng et al. (2013)	5,280,445
						Sun et al. (2019)	
	55	Luteolin-7-O-glucoside	C_21_H_20_O_11_	None	Root	Peng et al. (2013)	5,280,637
						Sun and wang. (2015)	
	56	Luteolin-7-glucuronide	C_21_H_18_O_12_	Ethanol, Impregnation extraction	Rhizome	Zhang et al. (2020a)	5,488,307
*Flavan-3-ols*	57	Catechin	C_15_H_14_O_6_	Water, Reflux	Root, rhizome	Nawrot-Hadzik et al. (2019)	9,064
	58	Catechin-3-O-gallate	C_22_H_18_O_10_	Water, Reflux	Root	Nawrot-Hadzik et al. (2019)	6,419,835
	59	Epicatechin	C_15_H_14_O_6_	Water, Reflux	Root, rhizome	(Nawrot-Hadzik et al., 2019)	72,276
						Fu et al. (2015)	
	60	Epicatechin-3-O-gallate	C_22_H_18_O_10_	70% Aqueous acetone, Reflux	Rhizomes, leaf	Nawrot-Hadzik et al. (2019)	107,905
						Liu et al. (2015)	
						(Bensa et al., 2020)	
	61	(+)-Catechin-5-O-glucoside	C_21_H_24_O_11_	Water, Reflux	Root, rhizome	Nawrot-Hadzik et al. (2019)	44,257,081
						Fu et al. (2015)	
						Yu et al. (2019)	
	62	(-)-Epicatechin-3-O-curvulin	C_25_H_22_O_10_	Methanol, Ultrasonic	Root, rhizome	Liu et al. (2015)	—
	63	(-)-Epicatechin-3-O-(E)-Caffeate	C_24_H_20_O_9_	Methanol, Ultrasonic	Root, rhizome	Liu et al. (2015)	—
*Procyanidins*	64	Procyanidins B1	C_30_H_26_O_12_	None	Fruit	Jug et al. (2021)	—
	65	Procyanidins B2	C_30_H_26_O_12_	None	Fruit	Jug et al. (2021)	—
	66	Procyanidins B3	C_30_H_26_O_12_	None	Fruit	Jug et al. (2021)	—
	67	Procyanidin B-5-3′-O-gallate	C_30_H_26_O_13_	Water, Decocting method	Fruit, flower, branch	Fu et al. (2015)	107,876
	68	Procyanidin C-13,3′,3″-tri-O-gallate	C_66_H_50_O_30_	30% Ethanol, Reflux	Fruit	Wang et al. (2015b)	117,772–85-7
*Flavanones*	69	Hesperetin	C_16_H_14_O_6_	Methanol, Reflux	Flower	Sun and wang. (2015)	72,281
	70	Hesperidin	C_28_H3_4_O_15_	Nhexane-ethyl acetate-ethanol-water (1:6:3:6, v/v/v/v), Reflux	Flower	Sun and wang. (2015)	—
*Isoflavones*	71	Genistein	C_15_H_10_O_5_	Methanol, Ultrasonic	Flower	Sun and wang. (2015)	5,280,961
**Phenylpropanoids**							
*Coumarins*	72	Coumarin	C_10_H_6_O_2_	90% Ethanol, Reflux	Root, rhizome	Yang et al. (2017)	323
	73	7-Hydroxy-4-methoxy-5-methylcoumarin	C_11_H_10_O_4_	Aqueous acetone, Reflux	Root, rhizome	Sun and wang. (2015)	5,318,268
	74	Polyisocoumarin	C_22_H_22_O_11_	80% Ethanol, Reflux	Air-dried and powdered rhizomes	Park et al. (2018)	—
*Simple phenylpropanoids*	75	Neochlorogenic acid	C_16_H_18_O_9_	Methanol, Reflux	Leaf	KurIta et al. (2016)	5,280,633
	76	Chlorogenic acid	C_16_H_18_O_9_	Methanol, Reflux	Root, flower	Peng et al. (2013)	1,794,427
	77	Caftaric acid	C_13_H_12_O_9_	Water, Reflux	Root	Park et al. (2018)	6,440,397
*Phenylpropanoid Disaccharide Esters*	78	Hydropiperoside	C_39_H_40_O_17_	Dlchloromethane, Ultrasonic	Rhizomes	Nawrot-Hadzik et al. (2018)	10,350,284
	79	Tatariside B	C_44_H_46_O_20_	70% Aqueous acetone, Ultrasonic	Air-dried and powdered rhizomes	Nawrot-Hadzik et al. (2018)	102,450,498
	80	Vanicoside A	C_41_H_42_O_18_	70% Aqueous acetone, Ultrasonic	Air-dried and powdered rhizomes	Nawrot-Hadzik et al. (2018)	10,724,147
	81	Vanicoside B	C_41_H_42_O_18_	70% Aqueous acetone, Ultrasonic	Air-dried and powdered rhizomes	Nawrot-Hadzik et al. (2018)	10,724,147
*Lignin*	82	Sodium (-)-lyoniresinol-2a-sulfate	C_22_H_27_O_11_SNa	60% Aqueous acetone, Ultrasonic	Root	Xiao et al. (2002)	—
	83	Sodium (+)-isolaricireinol-2a-sulfate	C_20_H_23_O_9_SNa	60% Aqueous acetone, Ultrasonic	Root	Xiao et al. (2002)	—
**Organic acids**						
	84	Tryptophan	C_11_H_12_N_2_O_2_	60% Aqueous acetone, Ultrasonic	Root	Xiao et al. (2002)	6,305
	85	2,6-Dihydroxy-bezoic acid	C_7_H_6_O_4_	60% Aqueous acetone, Ultrasonic	Root	Xiao et al. (2002)	—
	86	Citric acid	C_6_H_8_O_7_	Ethanol, Reflux	Tender stem, leaf	Zhang (1999)	311
	87	Tartaric acid	C_4_H_6_O_6_	Ethanol, Reflux	Tender stem, leaf	Zhang (1999)	875
	88	Hydroxysuccinic acid	C_4_H_6_O_5_	Ethanol, REflux	Tender stem, leaf	Zhang (1999)	525
	89	Oxalic acid	C_2_H_2_O_6_	None	Tender stem	Zhang (1999)	971
	90	4-Hydroxyacetophenone	C_8_H_8_O_2_	Methanol, Reflux	Flower	Sun and wang. (2015)	7,469
	91	Gallic acid	C_7_H_6_O_5_	Water, Decocting method	Root	Xiao et al. (2002)	370
	92	Protocatechuic acid	C_7_H_6_O_4_	Acetone, Ultrasonic	Root	(Wang 1984)	72
	93	Oleanolic acid	C_30_H_48_O_3_	95% Ethanol, Reflux	Root, rhizome	(Zuo et al., 2020)	10,494
Others							
	94	β-Sitosterol	C_29_H_50_O	Methanol, Ultrasonic	Root, rhizome, flower	Sun and wang. (2015)	521,199
						Jin et al. (2009)	
	95	2,5-Dimethy-7-hydroxy chromone	C_11_H_10_O_3_	Acetone, Ultrasonic	Root	(Wang 1984)	—
	96	Isotachoside	C_13_H_18_O_8_	60% Aqueous acetone, Ultrasonic	Root	(Xiao et al., 2003)	—
	97	Tachioside	C_13_H_18_O_8_	60% Aqueous acetone, Ultrasonic	Root	Xiao et al. (2002)	11,962,143
	98	5,7-Dihydroxy-1(3H)-isobenzofuranone	C_8_H_6_O_4_	None	Root	Peng et al. (2013)	11,062,751
	99	1-(3′,5′-Dihydroxyphenyl)-2-(4″- hydroxyphenyl)-ethane-1,2-diol	C_14_H_14_O_5_	60% Aqueous acetone, Ultrasonic	Root	Xiao et al. (2002)	11,139,908
	100	Sodium 3,4-dihydroxy-5-methoxybenzoic acid methyl ester-4-sulfate	C_9_H_9_O_5_SO_3_Na	60% Aqueous acetone, Ultrasonic	Root	Xiao et al. (2002)	—
	101	5-Hydroxymethyl-7-hydroxy-2-methylchromone	C_11_H_10_O_4_	Methanol, Reflux	Root	Kimura et al. (1983b)	—
	102	Torachrysone	C_14_H_14_O_4_	Methanol-water (80:20, v/v), Ultrasonic	Leaf	Wang et al. (2019c)	5,321,977
	103	1-(3-O-β-D-glucopyranosyl-4,5-dihydroxyphenyl)-ethanone	C_14_H_18_O_9_	60% Aqueous acetone, Ultrasonic	Root	Xiao et al. (2002)	—
	104	Vitamine C	C_6_H_8_O_6_	None	Tender stem	Zhang (1999)	—
	105	Daucosterol	C_35_H_60_O_6_	Methanol, Ultrasonic	Flower	Sun and wang. (2015)	5,742,590
	106	Torachrysone-8-O-(6′-acetyl)-glucoside	C_22_H_26_O_10_	Methanol-water (80:20, v/v), Ultrasonic	Leaf	Wang et al. (2019c)	—
	107	Torachrysone-8-O-β-D-glucoside	C_20_H_24_O_9_	Methanol-water (80:20, v/v), Ultrasonic	Leaf	Wang et al. (2019c)	—
	108	Ambrettolide	C_16_H_28_O_2_	95% Ethanol, Reflux	Root, rhizome	Jin et al. (2009)	5,365,703
	109	Galloyl-glucose	C_13_H_14_O_10_	70% Aqueous acetone, Reflux	Root, rhizome	Yu et al. (2019)	—
	110	5,7-Dimethoxyphthalide	C_10_H_10_O_4_	70% Ethanol, Reflux	Root	Lin et al. (2010a)	7,314,397

### 4.1 Quinones

**FIGURE 3:**
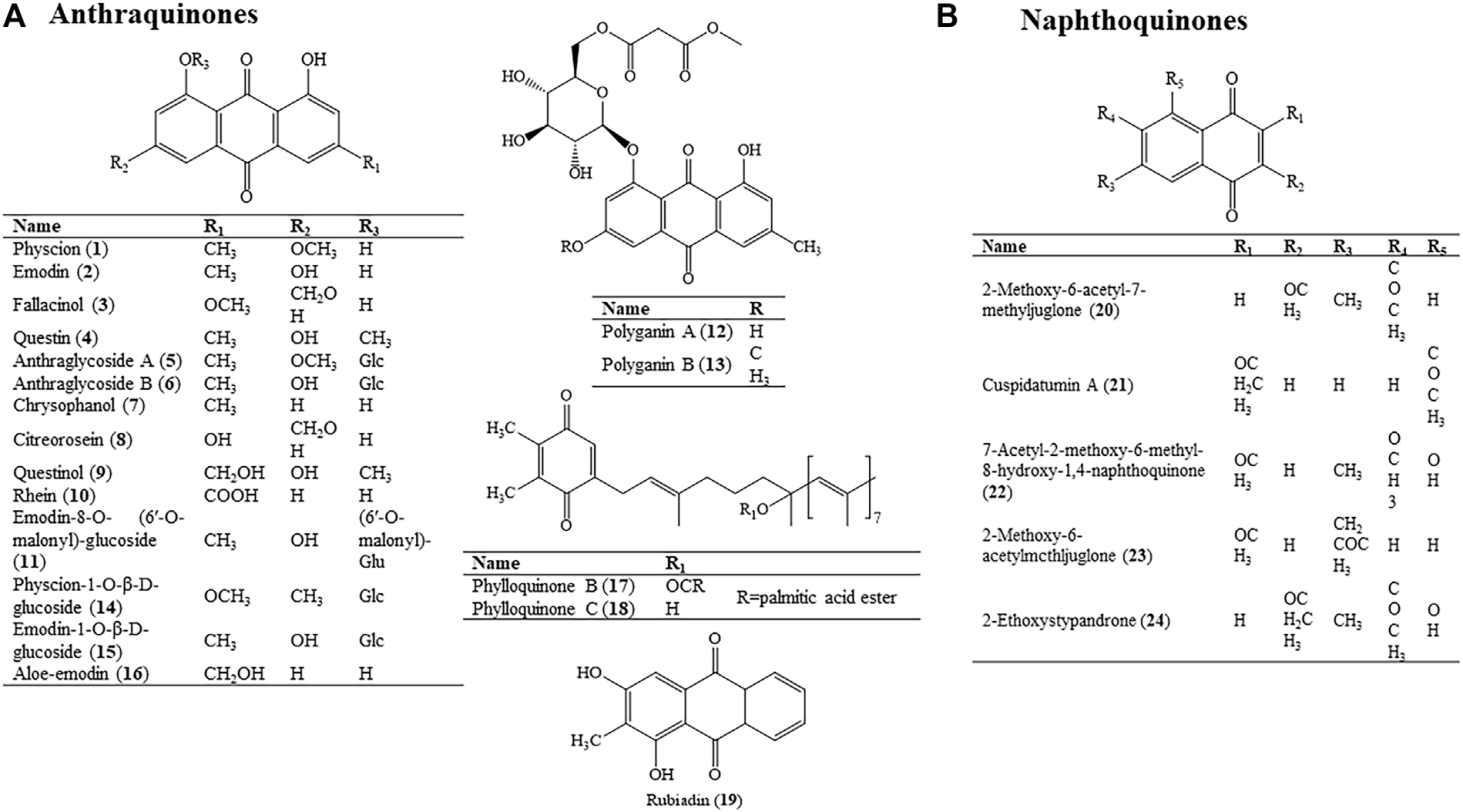
Molecular structure of quinones **(A)**. Anthraquinones; **(B)**. Naphthoquinones in HZ.

Up to now, quinones and their derivatives have been isolated and identified (Peng et al., 2013). These structurally unique quinones are classified into anthraquinones and naphthoquinones (Figure 3).

#### 4.1.1 Anthraquinones

Anthraquinones in HZ are mainly monoanthracene nuclei, among which the emodin type is the most common one, including physcion (1), emodin (2), questin (4), fallacinol (3), chrysophanol (7), rhein (10), citreorosein (8), questinol (9), aloe-emodin (16), and so on. Most of the components of anthraquinones are derived from roots and rhizomes, and a small part is derived from flowers and leaves. The article found that rhein (10) had the strongest inhibitory effect, followed by emodin (2), aloe-emodin (16), and chrysophanol (7). From the structural point of view, the mother nuclei of rhein (10), emodin (2), aloe-emodin (16), and chrysophanol (7) are the same, and the benzene ring has carboxyl, hydroxyl, and hydroxymethyl functional groups, respectively. Their polarity is carboxyl, hydroxyl, hydroxymethyl, and methyl in order. Therefore, we speculate that the influence of substituents on the anti-microbial efficacy may be related to their polarity; the stronger the polarity, the more powerful the antimicrobial activity (Wang J. et al., 2010). Similarly, different substitutions of functional groups can affect the anti-angiogenic activity; chrysophanol (7), emodin (2), and physcion (1) possess a methyl group at the C-3 position and only differ from one another at the C-6 position. Chrysophanol (7) with no substitution and physcion (1) with a methoxy group substitution did not exhibit any anti-angiogenic activity in the research results, while emodin (2) with a hydroxyl group at the C-6 position showed high activity. On the other hand, aloe-emodin (16), chrysophanol (7), and rhein (10) have no substitution at the C-6 position, but the differences in oxidation state of the methyl group or the absence of substitution at the C-3 position led to dramatic differences. Among them, rhein (10) with a carboxylic group displayed the strongest anti-angiogenic activity. Due to their planar chemical structure, the C-3 and C-6 positions of anthraquinones can be convertible in the emodin type. Therefore, we speculate that the structural characteristics of these three anthraquinones, acidic substitution with a phenolic or carboxylic group at C-3 or C-6 positions, or polar, hydrophilic substitution with a hydroxymethyl group at the C-3 position may contribute to the anti-angiogenesis potency (He et al., 2009).

#### 4.1.2 Naphthoquinones

There are a few naphthoquinones in HZ, such as 2-Methoxy-6-acetyl-7-methyljuglone (20), cuspidatumin A (21), 7-acetyl-2-methoxy-6-methyl-8-hydroxy-l, 4-naphthoquinone (22), and 2-Methoxy-6-acetylmcthljuglone (23).

### 4.2 Aromatic Hydrocarbons

**FIGURE 4:**
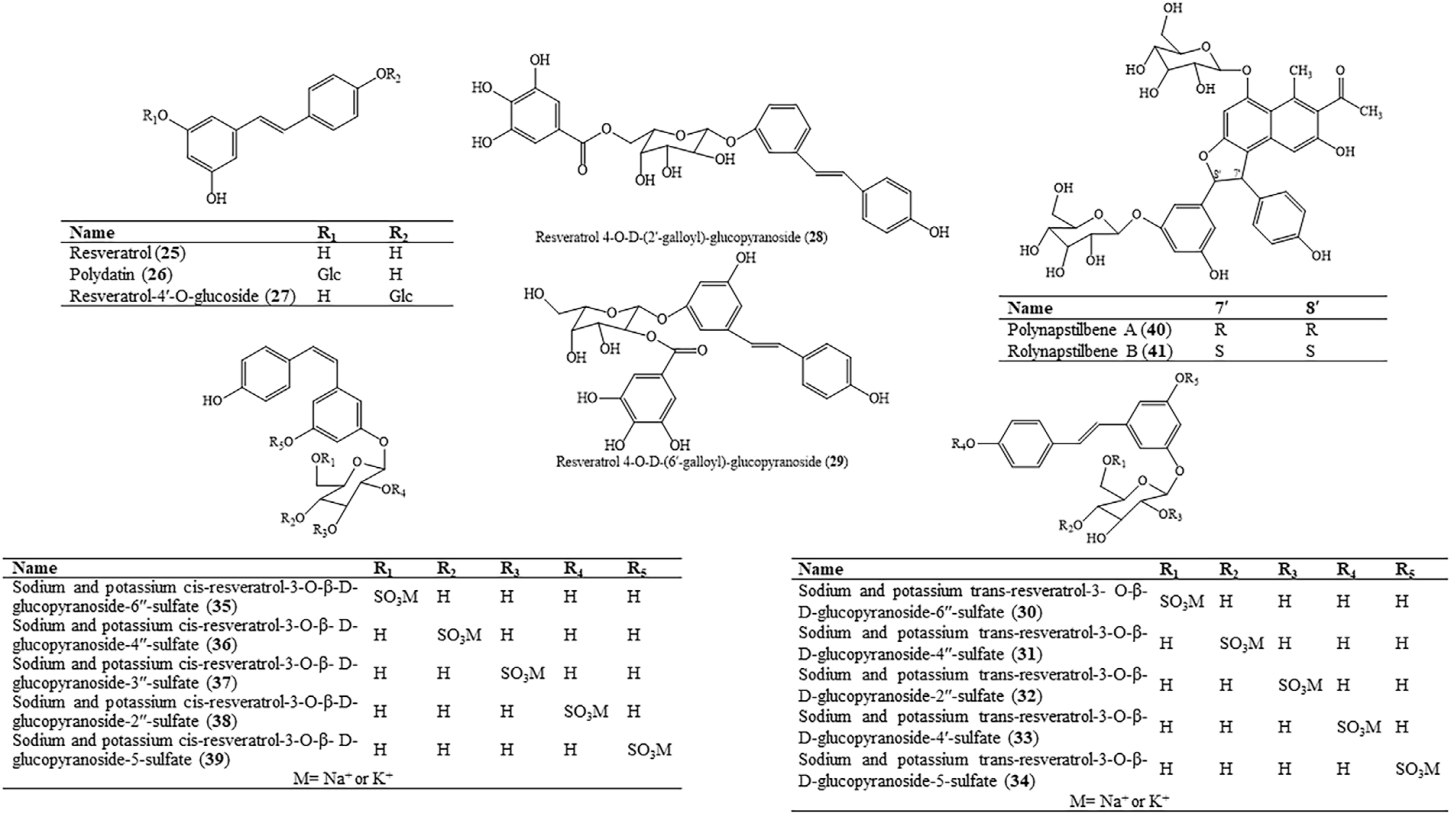
Molecular structure of aromatic hydrocarbons in HZ.

Stilbenes are discovered in only a few higher degrees of plant species via the general phenylpropanoid pathway. Stilbenes are other characteristic components of HZ and mostly distributed in the underground roots and rhizomes. Resveratrol (25) and resveratrol-3-O-glucoside piceid were isolated and identified from HZ in 1963 (Zhang YT. et al., 2020). Resveratrol-3-O-glucoside, also known as polydatin (26), is one of the index components of polydatin (26) specified in ChP. (Zhang YT. et al., 2020) (Figure 4).

Resveratrol (25) and polydatin (26), tow res glucosides, have been established to have beneficial effects on anti-carcinogenic effects (Soleas et al., 2001), inhibition of platelet aggregates (Orsini et al., 1997), anti-oxidation activity (Brohan et al., 2011), and so on*.* Polydatin (26) has the molecular structure of 3,4′,5-trihydroxystilben-3-β-D-mono-D-glucoside, which is quite similar to trans-resveratrol. The only difference between them is that polydatin (26) has a glucoside group at position C3 (Liu et al., 2017) and the presence of a glycosilic group in the polydatin (26) molecule allows to resist oxidation, prolongs its half-life, and increases its solubility (Regev-Shoshani et al., 2003). The hydroxyl radical can destroy almost all kinds of macromolecules including carbohydrates, nucleic acids, lipids, and amino acids. The hydroxyl radical is related to chronic health problems like cancer, arthrosclerosis, and ageing. Mechanisms for scavenging hydroxyl radicals for the protection of cellular structures include endogenous anti-oxidants such as melatonin and glutathione and dietary anti-oxidants. *In vitro* studies (Su et al., 2013) have shown that resveratrol (25) and polydatin (26) exhibited the capacity of scavenging hydroxyl radicals. Polydatin (26) showed higher scavenging activity against hydroxyl radicals than resveratrol (25) did. When the scavenging rate was 12%, the concentration of resveratrol (25) was 0.1 mmol/L and the concentration of polydatin (26) was lower than 0.05 mmol/L, while the concentration of vitamine C (104) was more than 0.113 mmol/L. This indicated that resveratrol (25) and polydatin (26) had higher hydroxyl radical scavenging capacity than vitamine C (104) at low concentrations. This is because the glycoside of polydatin (26) can enhance its anti-oxidant effect. Recently, many studies have researched the metabolism of polydatin (26) or resveratrol (25) in the body, showing that the mutual transformation between polydatin (26) and resveratrol (25) (Zhang WT. et al., 2008) (Zhou et al., 2009) can keep balance and they both have the ability of anti-oxidative stress *in vivo*, and polydatin (26) has a better effect than resveratrol (25), which may be related with its better oral absorption (Wang HL. et al., 2015). Therefore, we guess that the glycosilic group can enhance the oral absorption of polydatin (26). Furthermore, the polydatin (26) amount results to be more abundant than resveratrol (25) in the plants (Chen et al., 2014) (Xie et al., 2014) (Peng et al., 2015). It indicates that polydatin (26) might be a supplement of resveratrol (25) on clinical use.

### 4.3 Flavonoids

**FIGURE 5:**
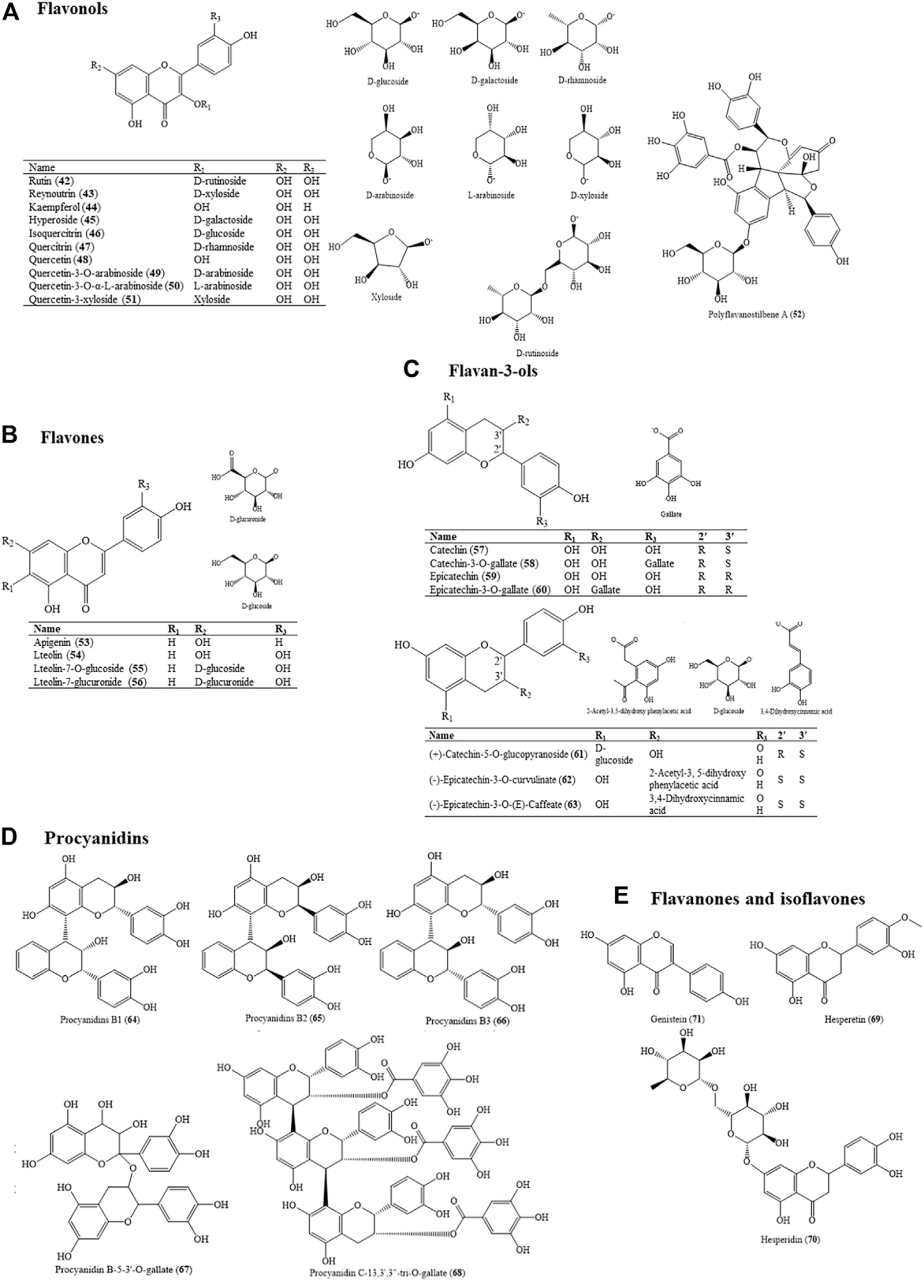
Molecular structure of flavonoids **(A)**. Flavonols, **(B)**. Flavones, **(C)**. Flavan-3-ols, **(D)**. Procyanidins, and **(E)**. Flavanones and isoflavones in HZ.

Flavonoids are distributed in all plants of HZ, especially in the roots and leaves. Flavonoid glycosides generally existed in flowers, fruits, and leaves, while free camphons mainly existed in stems and roots (Zhao 2017). According to different oxidation conditions and distinctive types and positions of substituents, flavonoids in HZ are mainly divided into four types: flavonols, flavonoids, flavan-3-ols, and procyanidins. Among the extraction techniques of flavonoids from HZ, Hu used the orthogonal design experiment to optimize ultrasonic extraction of total flavonoids (Hu et al., 2013). Under the optimal technological conditions, the extraction rate of total flavonoids could reach at 7.68% (Figure 5).

#### 4.3.1 Flavonols

Kuznetsova obtained some polyphenol compounds from the roots, including quercetin (48), quercetin-3-O-arabinoside (49), quercitrin (47), isoquercitrin (46), and hyperoside (45), which belongs to flavonols (Fukuyama et al., 1988). The leaves of HZ also contain quercetin-3-xyloside (51)[5], quercetin-3-O-α-L-arabinoside (50), and reynoutrin (43) (Zhang 1999) (Medica 1999). Sun isolated flavonols from the methanol extract of flowers, including rutin (42), kaempferol (44), and quercetin (48) (Sun et al., 2015). Rutin (42), kaempferol (44), and quercetin (48) play important roles in anti-bacterial and anti-viral activities, among which rutin (42) and quercetin (48) have strong cardiovascular activity. According to Zhou [8], the diuretic activity of HZ is flavonol glycosides; the representative compounds are isoquercitrin (46) and quercein-3-O-arabinoside (49) (Zhou 1986). Polyflavanostilbene A (52) was isolated from the rhizomes of HZ, which showed strong inhibitory activity against α-glucosidase with an IC50 value of 17.7 Mm (Li et al., 2013).

#### 4.3.2 Flavones

Luteolin (54) and its derivatives, mostly in the form of glycosides in this plant, have pharmacological effects such as anti-tumor, anti-inflammation, anti-virus, anti-oxidation, immune regulation, and so on (Mo et al., 2021). Flavonoid anti-oxidants with 2- or 3-phenylchroman structures such as apigenin (53) and luteolin (54) may reduce coronary disease and cancer (Griffiths et al., 2016). Compared with other flavonoids [quercetin (48) and kaempferol (44)], apigenin (53) has the characteristics of low toxicity and no mutagenicity (Fu et al., 2020).

#### 4.3.3 Flavan-3-Ols

Flavane-3-alcohols, also as known as catechin (57), are widely found in plants. The compounds C-2 and C-3 are chiral carbons, and the flavane-3-alcohol configurations of HZ are generally (2R, 3S) and (2R, 3R). There are some catechin (57) and its derivatives (Kimura et al., 1983), which are natural lipid anti-oxidants and can scavenge free radicals produced by the human body to protect the cell membrane. However, catechin (57) is unstable and easily oxidized to form polymers (Zhang P. et al., 2020). In addition, (+)-Catechin-5-O-glucoside (61) and epicatechin-3-O-gallate (60) were isolated from the rhizomes of HZ by a method as follows: 25 g of HZ was immersed in 500 ml of distilled water for 1 h and heated with a heating mantle (Fu et al., 2015). Liu found (-)-Epicatechin-3-O-curvulinate (62) and (-)-Epicatechin-3-O-(E)-Caffeate (63) by using air-dried and powdered HZ with ethanol–water under reflux for 2 h (Liu et al., 2015).

#### 4.3.4 Procyanidins

Statistical analysis demonstrated that procyanidins should be considered as important contributors to the total anti-oxidant capacity (Nawrot-Hadzik et al., 2019). Procyanidins B1 (64), procyanidins B2 (65), and procyanidins B3 (66) were isolated from the bark of the rhizomes of HZ by off-line multidimensional high-performance thin-layer chromatography (Jug et al., 2021). Procyanidins B1 (64) and procyanidins B2 (65) are the first detection of some compounds in the bark of Japanese knotweed rhizomes and Japanese knotweed rhizomes in general [14]. A study showed that three enantiomers of procyanidin B were found in the extracted ion chromatograms of ions (EICs) at 577.13 m/z (Fu et al., 2015). Wang had isolated procyanidin C-13,3′,3″-tri-O-gallate (named as REJ-C1G3) (68) from HZ that could activate the human immunodeficiency virus (HIV) in latently infected Jurkat T-cells (Wang C. et al., 2015). Researchers confirmed the presence of procyanidins with a high degree of polymerization, up to decamers, in the rhizomes of HZ. The ethyl acetate fractions, rich in proanthocyanidins, also in the galloylated form, were the most active in all anti-oxidant tests (Nawrot-Hadzik et al., 2019).

#### 4.3.5 Flavanones and Isoflavones

Three compounds, hesperetin (69), hesperidin (70), and genistein (71), were isolated and identified from the extraction of HZ’s flowers. New findings (Parhiz et al., 2015) showed that the anti-oxidant activity of hesperidin (70) and hesperetin (69) was limited to its radical scavenging activity, and it augmented the anti-oxidant cellular defenses via the extracellular regulated protein kinases (ERKs) and the transcription factor nuclear factor erythroid 2 (NF-E2)-related factor 2 (Nrf2) signaling pathway as well. Genistein (71) is an isoflavone present and is known to have multiple molecular effects, such as the inhibition of inflammation, promotion of apoptosis, and modulation of steroidal hormone receptors and metabolic pathways (Mukund et al., 2017).

In addition, a study discovered that flavonoids from the roots of HZ could inhibit the activity of lipoxygenase in soybean and Maojian tea (Wu et al., 2020). The animal experiment showed that flavonoids of HZ had a good hypoglycemic effect *in vivo* by the postprandial glucose test of normal mice and the continuous administration of alloxan in diabetic mice (Zhou et al., 2007). A clear correlation had also been found between the anti-microbial activity and the flavonoid content of the plant ethanol extracts by the test against fungi, yeast, and Gram-negative and Gram-positive bacteria (Zhang L. et al., 2013). In conclusion, the pharmacological activity of flavonoids from HZ has obviously a good prospect of development and application.

### 4.4 Phenylpropanoids

**FIGURE 6:**
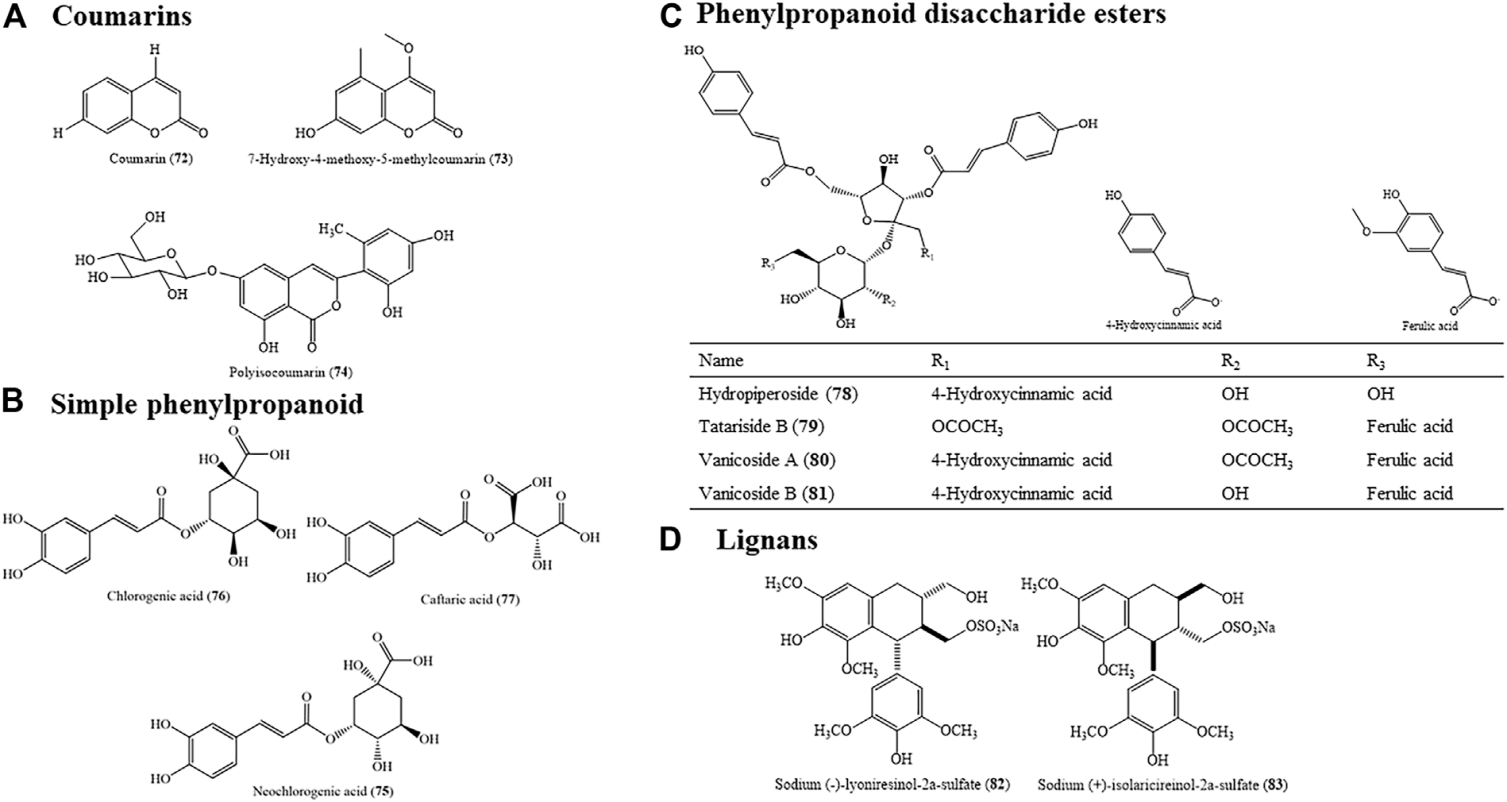
Molecular structure of phenylpropanoids: **(A)** coumarins, **(B)** simple phenylpropanoid, **(C)** phenylpropanoid disaccharide esters, and **(D)** lignans in HZ.

According to different conditions and positions of substituents, phenylpropanoids in HZ are mainly divided into four types: coumarins, simple phenylpropanoids, lignans, and phenylpropanoid disaccharide esters with a C6-C3 carbon frame structure. The chemical structures of phenylpropanoids are shown in Figure 6.

#### 4.4.1 Coumarins

In 1983, researchers isolated 7-hydroxy-4-methoxy-5-methylcoumarin (73) from the roots of HZ by using aqueous acetone. Later, another coumarin compound named coumarin (72) was isolated from this plant (Yang et al., 2017). Coumarin (72) and derivates are proposed as lipid-lowering agents due to its broad pharmacological activities, mainly implicated in vasodilator and anti-oxidant effects (Tejada et al., 2017). A new isocoumarin derivative, polyisocoumarin (74), was isolated from HZ. The cytotoxicity activity and protein tyrosine phosphatase 1B (PTP1B) inhibitory activity of these compounds was estimated, and none of them exhibited activities at a concentration of 10 μmol/L.

#### 4.4.2 Simple Phenylpropanoid

Simple phenylpropanoid is a common aromatic compound in traditional Chinese medicine. According to the different functional groups of three carbon chain C3 and the compounds extracted from HZ, it can be further divided into phenylpropanoic acid. As shown by 1,1-diphenyl-2-picrylhydrazyl (DPPH) radical and superoxide anion scavenging assays, the contributions of neochlorogenic acid (75) as an anti-oxidant were 16.5 and 36.5%, respectively, suggesting that neochlorogenic acid (75) is the predominant anti-oxidant in leaves by the methanol extract of fresh HZ (KurIta et al., 2016). Chlorogenic acid (76), which showed the initial rates of DPPH radical scavenging with 0.27 ± 0.06 mmol/min in polyphenol compounds, was found to be one of the polyphenolic components in the leaves and stems of the plant (Kirino et al., 2012).

#### 4.4.3 Phenylpropanoid Disaccharide Esters

Hydropiperoside (78) and tatariside B (79) were reported for the first time in HZ by a reversed-phase high-performance liquid chromatography method with a diode array detector and time-of-flight mass spectrometry (Nawrot-Hadzik et al., 2018). Vanicoside A (80) and vanicoside B (81) were quantified using the validated method (Nawrot-Hadzik et al., 2018).

#### 4.4.4 Lignans

Additionally, two lignan sulfates were isolated from an aqueous extract of this plant, including sodium (-)-lyoniresinol-2a-sulfate (82) and sodium (+)-isolaricireinol-2a-sulfate (83) (Xiao et al., 2002). They exhibited no inhibition of lipid peroxidation and no cytotoxic and DNA cleavage activities.

### 4.5 Organic Acids

**FIGURE 7:**
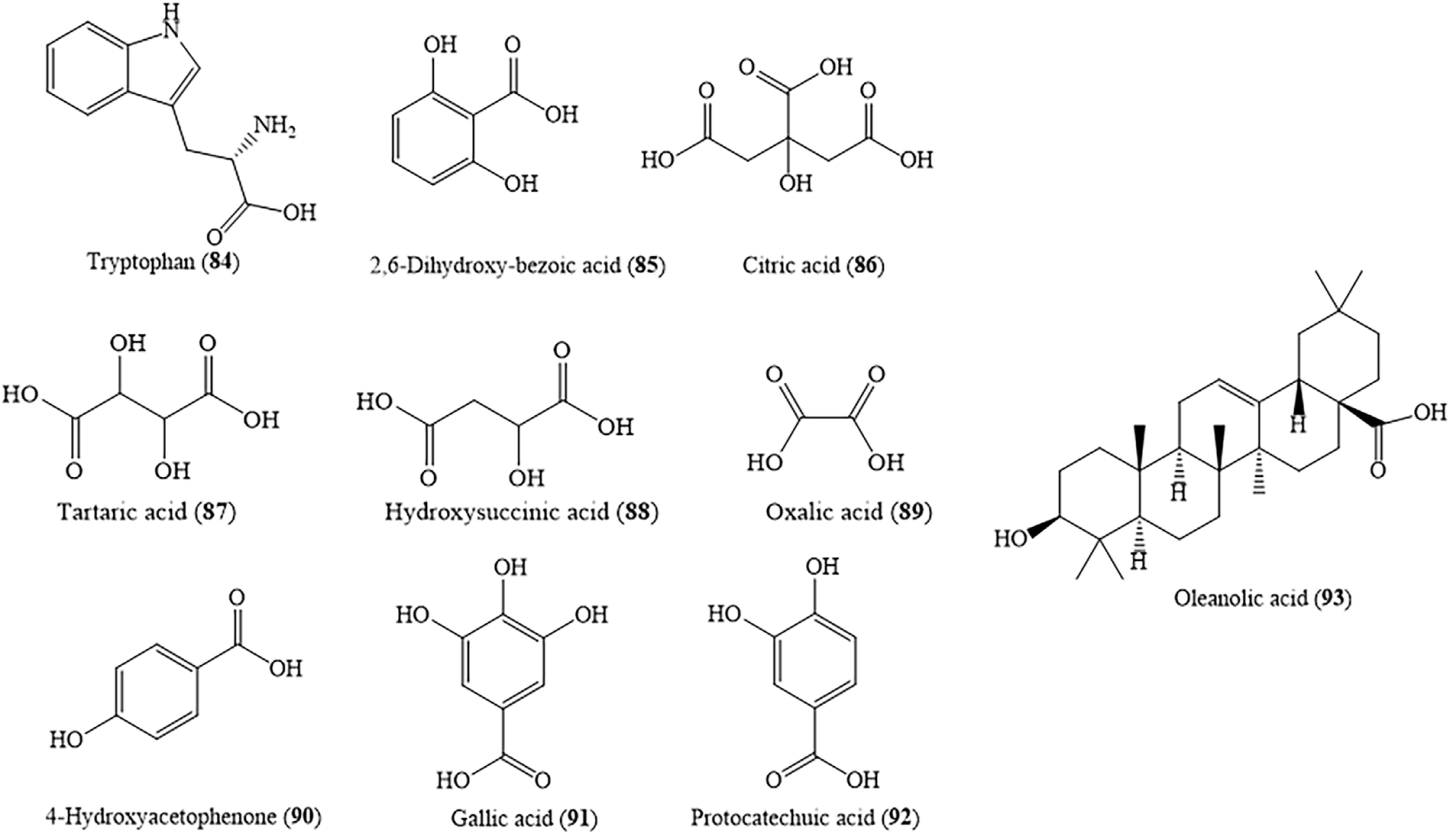
Major organic acids presenting in HZ.

HZ contains organic acids in its roots, tender stems, and leaves, but there is also a part of it in the flower. So far, at least 10 organic acids have been obtained from this herb. They can be separated into phenolic acids, fatty acids, and so on*.* Gallic acid (91) and protocatechuic acid (92) are organic acids which had been studied, and they are significant active substances of HZ. Xiao isolated and identified gallic acid (91) from the roots of HZ by water extraction for the first time (Xiao et al., 2002). It has been shown that gallic acid (91) and protocatechuic acid (92) not only could reduce blood glucose (Ibitoye et al., 2018) but also could prevent cardiovascular diseases including atherosclerosis (AS), coronary artery disease, and so on (Adigun et al., 2016). Both gallic acid (91) and protocatechuic acid (92) can pierce through the active-site cleft of the N-ter (N-terminal) catalytic domain of human maltase-glucoamylase (hMGAM) as well as into a small number of non-active site clefts to lower blood sugar by forming hydrogen bonds (Alegbe et al., 2019). Taken as a whole, this study implies that gallic acid (91), with one more hydroxyl group on its phenolic ring, may display a higher level of inhibitory activity than protocatechuic acid (92) against hMGAM *in vivo* due to its increased hydrogen-bonding potency. The chemical structures of organic acids are shown in Figure 7, and the names of these compounds are listed in Table 3.

### 4.6 Carbohydrate

A polysaccharide was isolated from HZ, which has a molecular weight of about 6,000 and contains 38 monosaccharides, including D-glucose, D-galactose, sucrose, L-rhamnose, and L-arabinose, with a ratio of 28:4:4:1:1 (Sun et al., 2015) (Ouyang 1987). A result showed that polysaccharides including PCP-30, PCP-50, PCP-70, and PCP-90 were prepared by different ethanol-fractionated precipitation methods with about 30, 50, 70, and 90% concentrations from HZ, respectively (Wang J. et al., 2019). In the concentration range of 0.5–8 mg/ml, the DPPH radical scavenging activity and reducing power of the polysaccharides in the four groups were concentration-dependent, and the higher the concentration, the stronger the scavenging rate. Additionally, *in vitro* anti-oxidation and analgesic experiments showed that this polysaccharide had better anti-lipid peroxidation and analgesic effects (Wang 2009). Meanwhile, the effect of this polysaccharide on serum-related enzyme activity can enhance the spleen index. Three light-brown polysaccharides (PPA, PPB, and PPC) were obtained by water extraction and fractional alcohol precipitation from HZ, which had strong inhibitory activity against α-glucosidase with IC50 values of 114.82 mg/ml, 75.68 mg/ml, and 70.31 mg/ml (Liu XQ. et al., 2018). Another paper showed that the coarse polysaccharide (PP) in HZ had good α-glycosidase enzyme inhibition activity, while homogeneous polysaccharides had almost no α-glycosidase enzyme inhibition activity, indicating that the hypoglycemic activity of total PP is the result of the synergistic relationship with the HZ pigment and protein (Zhang 2017).

### 4.7 Others

**FIGURE 8:**
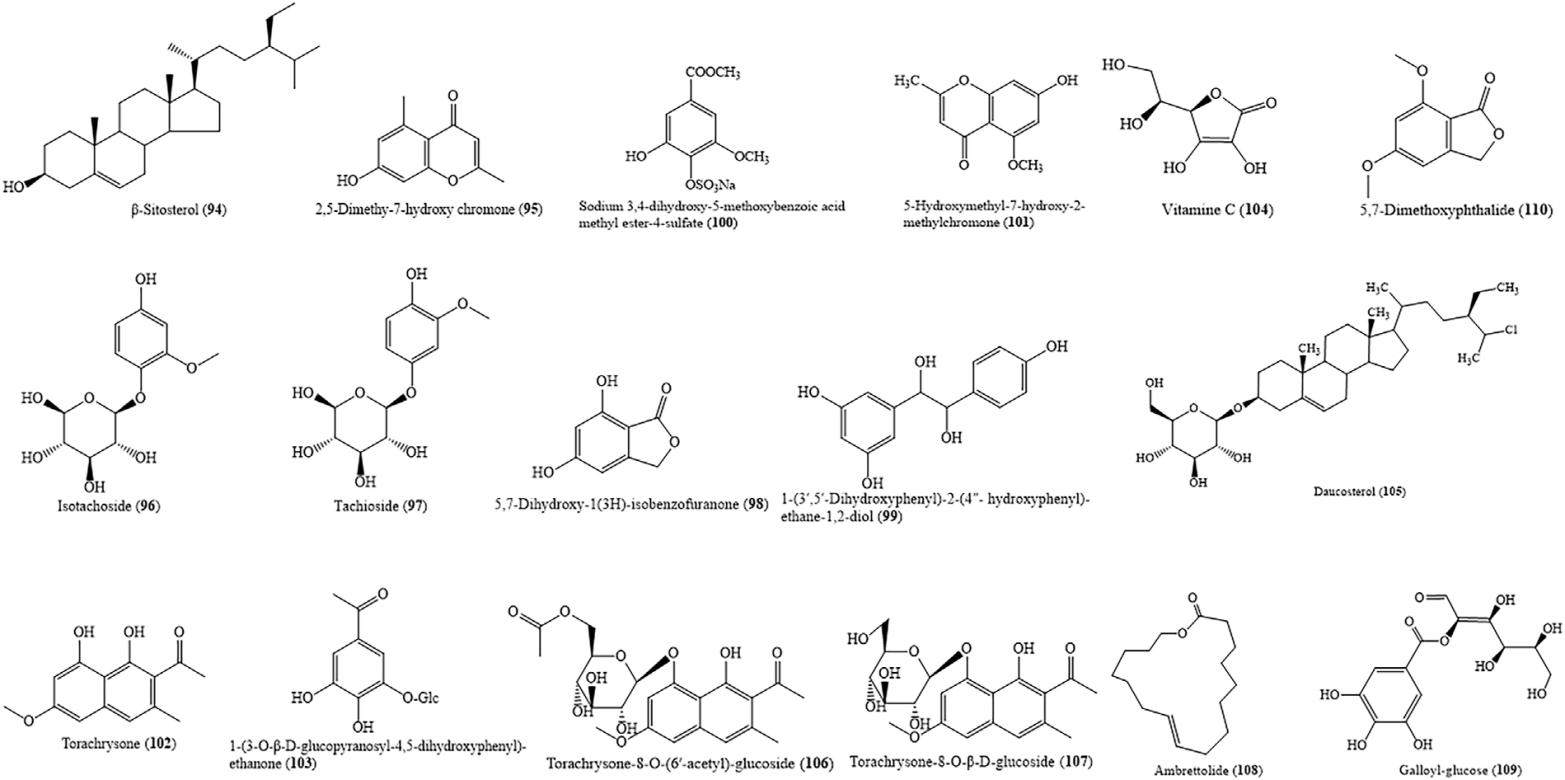
Major others presenting in HZ.

In addition to the above components, there are other ingredients in HZ including β-sitosterol (94), 2,5-dimethyl-7-hydroxy chromone (95), torachrysone (102), and so on. HZ is rich in the nutrients necessary for human life, such as daucosterol (105), vitamine C (104), and so on. All these ingredients join together to form a large and complex material basis of HZ. The chemical structures of these compounds are shown in Figure 8.

From the above, HZ mainly contains anthraquinones, stilbenes, and flavonoids. Among them, anthraquinones and stilbenes are the most important components in HZ, accounting for 39.54 and 24.34%. Emodin (2) and polydatin (26) are not only the quality control components stipulated in HZ according to the 2020 edition of ChP but also the representative monomer components of these two kinds of components. Anthraquinones and stilbenes are widely distributed in Polygonaceae, such as Rhei radix et rhizoma (called Dahuang, DH in Chinese) and Polygoni multiflori radix (called Heshouwu, HSW in Chinese). Studies (Tao et al., 2016) have shown that anthraquinones relieve constipation and lower blood lipid and blood pressure (Tao et al., 2016), while stilbenes have hepatoprotective and neuroprotective effects (Hang et al., 2016), which support that DH, HZ, and HSW (Wu 2012) could treat constipations and hepatic and gall diseases in clinics. However, as far as anthraquinones are concerned, 10 kinds of anthraquinones in DH can be detected and 6 kinds of anthraquinones can be detected in HZ, while only 4 kinds of anthraquinones can be detected in HSW by HPLC-UV (Li et al., 2020). Therefore, there are some differences in the treatment of constipation of these three kinds of traditional Chinese medicine in the clinical application. DH (Ji et al., 2019) (Yu WM. et al., 2019) (Wang et al., 2021a) is often used in the treatment of functional constipation which is characterized by abdominal distension and a few stools, the dry stool and difficult stool. HSW (Fang 2015; Yu WM. et al., 2019) can treat habitual constipation and can promote intestinal peristalsis and treat intestinal dryness and constipation due to blood deficiency. Zhang (Sun et al., 2019) reported that HSW contains bound anthraquinone derivatives, which can promote intestinal peristalsis and produce purgative effects. Through the treatment of 60 cases of functional constipation (Ni 2010) in the elderly with the compound HZ mixture, the data showed that the total efficiency was 91.7%. Accordingly, the efficacy of HZ (Yang B. et al., 2019) depends not only on a single component but also on the synergism of multiple components. The different types and contents of components as well as the interaction between components to produce new active ingredients will eventually lead to differences in the efficacy of different medicinal materials. Extracts obtained from this plant, fungi, or animals pose some unique challenges: they are multicomponent mixtures of active, partially active, and inactive substances, and the activity is often not on a single target.

## 5 Biological Activities

**FIGURE 9:**
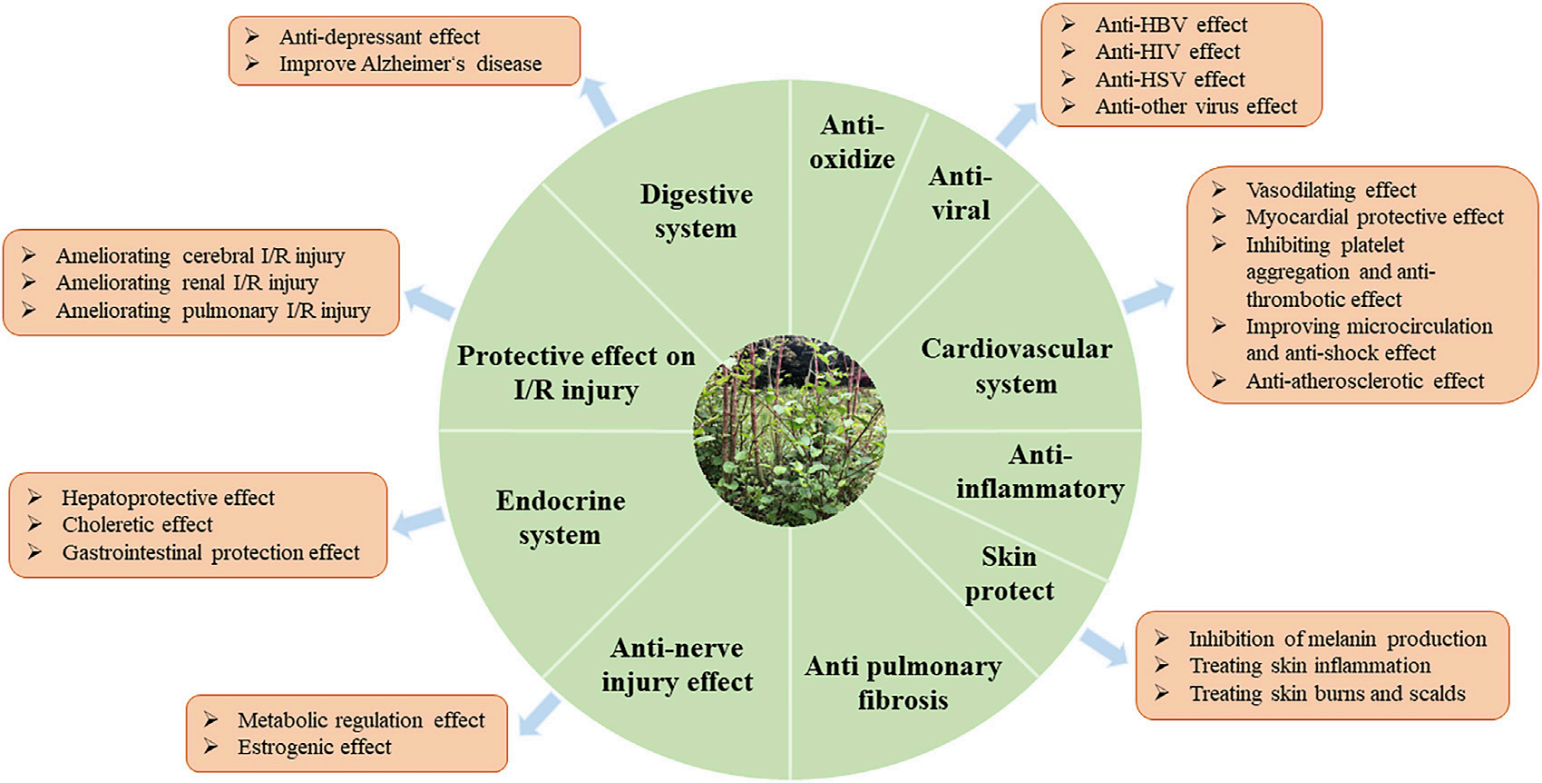
Biological activities of *Reynoutria japonica* Houtt.

**FIGURE 10:**
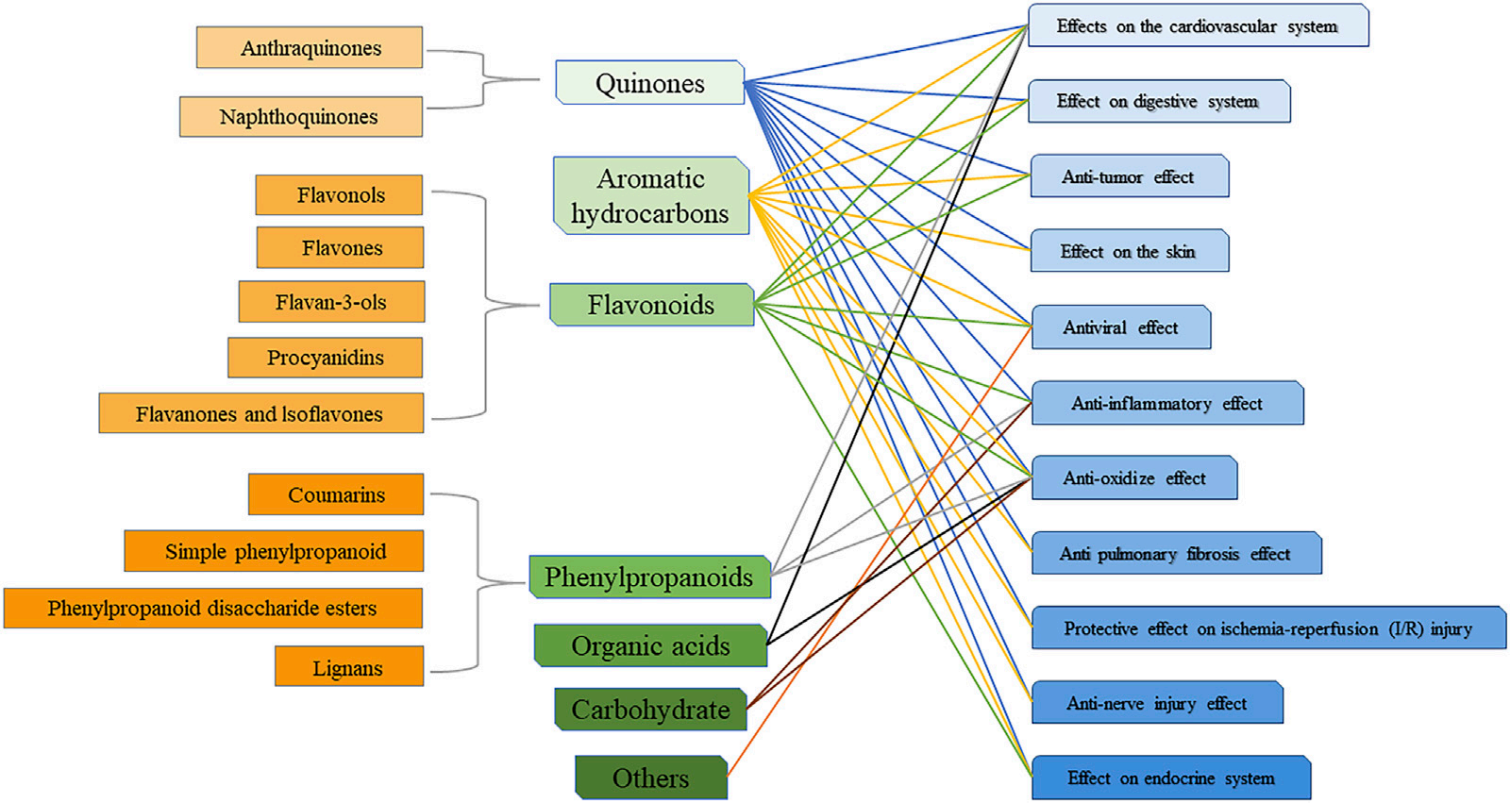
Chemical components and biological activities of *Reynoutria japonica* Houtt.

Modern pharmacological studies have shown that HZ has wide pharmacological activities such as cardiovascular, anti-tumor, anti-inflammatory, and anti-virus, protecting the liver and gallbladder, skin burns, and so on, which coincides with its traditional effects such as breaking blood, dispelling wind, relieving pain, heat clearing, detoxification, and converging sores (Figure 9). Among them, the pharmacological effects of expelling wind and relieving pain in HZ are closely related to its good anti-inflammatory and anti-oxidant effects. Extracts obtained from HZ pose some unique challenges: they are multicomponent mixtures of active, partially active, and inactive substances, and the activity is often not on a single target. A relation between the biological activites and components of HZ is presented in Figure 10.

### 5.1 Effects on the Cardiovascular System

In TCM, HZ has the potencies of breaking blood and dispersing blood stasis, and it is often used to treat women’s dysmenorrhea and so on. Modern pharmacological studies also show that HZ and its components have good effects of dilating blood vessels, anti-shock effects, and inhibiting platelet aggregation, and its blood-activating effect is widely used in cardiovascular diseases (Liao et al., 2012).

#### 5.1.1 Vasodilating Effect

Liu crushed HZ into coarse powder and fried in water, and then the filtrate was prepared into 2 × 103 mg/ml decoction. Then, the water decoction was prepared into six series of concentrations of 10–1, 3 × 10–1, 1, 3, 10, and 2 × 10 mg/ml, respectively. It was observed that HZ could increase the rate of the right atrium of guinea pigs and reduce the resting tension of rabbit vascular smooth muscles by dilating blood vessels. There was an obvious dose–effect relationship, and its effect might be related to the α-receptor and H1 receptor (Liu et al., 2008).

Polydatin (26) is the effective ingredient in HZ to dilate blood vessels. Wu used the cumulative concentration method (increment in 0.5 logarithmic units, 1 × 10–9∼3 × 10–5 mol/L) to observe the diastolic effect of polydatin (26) on the isolated thoracic aorta of Sprague–Dawley (SD) rats contracted by phenylephrine pretreatment. Experimental results showed that in the normal control group with an intact endothelium, polydatin (26) relaxed the rat thoracic aortic rings precontracted by phenylephrine in a concentration-dependent manner, and the maximum relaxation effect was (79.75 ± 8.34%) and the logarithm half-maximal inhibitory concentration (-logIC50) was (6.13 ± 1.55), which was significantly different from that in the endothelium-removed group (n = 6, *p* < 0.05). It suggested that the effect of polydatin (26) was endothelium-dependent (Wu Y. et al., 2014). Luo reported that polydatin (26) had a significant vasodilating and anti-hypertensive effect. 1.71 × 10–3 mol/L polydatin (26) could non-competitively inhibit the contraction of the isolated rabbit pulmonary artery by norepinephrine and shift the dose–effect curve of norepinephrine to the right. The results showed that the pulmonary artery was significantly dilated 10–20 min after polydatin 4.09 × 10–3 mol/L and 5.12 × 10–3 mol/L. At the same time, the effect of isoproterenol on the pulmonary artery was also observed. The action of isoproterenol with the pulmonary artery showed relaxation effects in 5min (Luo 1992).

Yang used the isolated rabbit aortic perfusion ring model and accumulated quercitrin (47) solution according to the concentration gradient so that the final concentration in the perfusate was 1 × 10–6, 3 × 10–6, 1 × 10–5, 3 × 10–5, 1 × 10–4, 3 × 10–4, and 1 × 10–3 mol/L to study the effect and mechanism of quercitrin (47) on isolated rabbit aortic rings. The results showed that quercitrin (47) had an endothelium-independent relaxation effect on the rabbit aorta, and its mechanism might be related to the activation of guanylate circulation in vascular smooth muscles (Wang X. et al., 2019).

#### 5.1.2 Myocardial Protective Effect

Myocardial necrosis caused by local ischemia and hypoxia is one of the main causes of myocardial infarction (MI)-induced heart failure. Reducing the myocardial infarction size (MIS) and improving myocardial functions have become the main therapeutic targets of drugs against MI (Wu et al., 2017a). In addition, myocardial ischemia often leads to oxidative stress and could be evaluated by using the lipid peroxide (LPO), malondialdehyde (MDA), and superoxide dismutase (SOD) levels, and some myocardial enzymes including creatine phosphokinase (CPK) and lactate dehydrogenase (LDH) were often used as indicators of myocardial injury evaluation. Studies have shown that HZ decoction could significantly enhance the contraction of the isolated heart (Fan et al., 2013). Yu established the model of myocardial infarction by permanent ligation of the left anterior descending coronary artery. After the establishment of the model, different doses of flavonoids extracted from HZ (FHZ) were given intragastrical once a day for 2 weeks. Experimental results showed that FHZ treatment could reduce serum cardiac troponin T (cTnT) levels and MIS values in mice with MI, indicating that FHZ had a therapeutic effect on MI. Moreover, FHZ decreased the serum levels of CPK, LPO, MDA, and LDH and increased the SOD level in MI mice, demonstrating that FHZ protected against MI by attenuating oxidative stress and decreasing the levels of myocardial enzymes (Yu HR. et al., 2019).

Furthermore, some scholars have found that polydatin (26) and resveratrol (25), the monomer components of HZ, also had protective effects on cardiomyocytes. In the acute myocardial infarction (AMI) rat model caused by ligation of the left anterior descending branch of the left coronary artery in SD rats, Le found that the levels of LDH, creatine kinase (CK), and CK isozyme (CK-MB) in the serum of AMI rats significantly decreased after the treatment of polydatin (26) (40 mg/kg) (*p* < 0.05), and the area of MI decreased significantly (*p* < 0.05). In addition, the messenger ribonucleic acid (mRNA), protein expression levels, protein expression levels of transcription factor nuclear factor-erythroid 2 (NF-E2)-related factor 2 (Nrf2) and Heme Oxygenase-1 (HO-1) in cardiomyocytes increased significantly (*p* < 0.05). Finally, they concluded that polydatin (26) could reduce cardiomyocyte injury after acute MI in rats by activating the Nrf2/HO-1 pathway (Lei et al., 2019). Zhang treated cardiac fibroblasts (CFs) with resveratrol (25) (50 μmol/L) and found that it could inhibit the secretion of CF collagen induced by transforming growth factor-β1 (TGF-β1) (*p* < 0.001), confirming that resveratrol (25) could downregulate microRNA-17 (miR-17) and regulate SMAD family member 7 (SMAD7) to inhibit TGF-β1-induced CF proliferation and collagen secretion (Zhang et al., 2018).

#### 5.1.3 Inhibiting Platelet Aggregation and Anti-thrombotic Effects

Thrombosis is a very complex pathophysiological process; its important pathogenetic factors are the mutual intercellular reactions among platelets, white blood cells, endothelial cells, and so on (Liu et al., 2012). Lv studied the effects of different extracts of HZ on promoting blood circulation and removing blood stasis. The water decoction extract, water extraction alcohol precipitation extract, and alkali water percolation alcohol precipitation extract were collected. After intragastric administration of high and low doses (110 g/kg, 50 g/kg) to mice for 7 days, blood was taken from the orbit, and then the plasma-activated partial thromboplastin time (APTT), prothrombin time (PT), and fibrinogen (FIB) content were measured. The results showed that the three extracts of HZ could prolong the time of APTT and PT and decrease the content of FIB, and the effect of water extract was more significant with the increase of dose (*p* < 0.001). In addition, the acute toxicity test showed that the dose of 50 g/kg and 100 g/kg HZ water decoction had no toxic reaction in mice (Lv et al., 2010).

In the rabbit platelet aggregation test *in vitro*, Chen found that each dose of polydatin (26) had a concentration-dependent inhibitory potency on rabbit platelet aggregation induced by arachidonic acid (AA) and adenosine diphosphate (ADP). In *in vivo* experiments, the rabbits were injected intravenously (5, 10, 20 mg/kg) with polydatin (26) according to the group, and then the blood was collected according to the time point *in vitro* experiments. They found that 0.5 h significantly inhibited the rabbit platelet aggregation induced by AA and ADP (Chen et al., 2005). Wang administered polydatin (26) (40, 80, 160 mg/kg) to rats by intragastric instillation. The animals were subcutaneously injected with adrenaline 1 day before blood collection, and the rats were immersed in ice water for 5 min to make an acute blood stasis model. The experimental results found that polydatin (26) showed an antagonistic action on thrombosis, which significantly reduced the fibrinogen content and platelet adhesive rate in acute blood-stasis model rats (Wang et al., 2004b).

#### 5.1.4 Improving Microcirculation and Anti-shock Effects

Polydatin (26), the active component of HZ, could significantly enhance the symptoms of heart failure in burn shock and hemorrhagic shock, strengthen myocardial contractility, and increase cardiac output (Fan et al., 2013). Wang used rat arterial bloodletting to reduce the blood pressure to 30 mmHg and maintained for 2 h to create a shock model and then intravenously injected polydatin (26) (30 mg/kg) *in vivo* for treatment. The experimental results showed that polydatin (26) could significantly prolong the survival time of severe shock rats. In the treatment of shocked rats with polydatin (26), the survival time of rats was significantly prolonged to 4.35 times that of the control group, and the 24 h survival rate was 5/8, which was significantly better than those of other treatment groups and control groups (*p* < 0.001) (Wang 2013).

#### 5.1.5 Anti-atherosclerotic Effects

AS is a common disease which seriously endangers people health. It is the main pathological basis of coronary heart disease, cerebrovascular disease, thromboembolic disease, and other ischemic cardio-cerebrovascular diseases. Studies have found that lipid metabolism disorders were the pathological basis of AS and polydatin (26), the main components of HZ, and could prevent AS by regulating the blood lipid metabolism.

Vascular endothelial dysfunction (ED) is the key to the pathogenesis of AS. Qin divided the experiment into the control group, high-fat group, different dose groups of HZ (9g/day, 3g/day, 1g/day), and the hyperlipidemic positive control group (L-arginine 4.22 g/day). It was found that the onset of endothelium-dependent dilation (EDD) disorder in the high-dose group was later, and the effect was similar to that in the high-fat positive control group. The levels of serum nitric oxide (NO), plasma endothelin-1 (ET-1), and nitric oxide synthase (NOS) activity in the HZ group decreased in a dose-dependent manner. The results showed that HZ could improve the function of the disordered NOS system and alleviate the pathological changes of AS in a dose-dependent manner, especially in the high-dose group (Qin et al., 2005). Ma made a rabbit AS model by feeding with high-fat diet. All groups were treated with HZ (2 g/day), polydatin (26) (15 mg/day), and emodin (2) (160 mg/day) except for the normal group and high-fat group. The outcome showed that there were differences in TC and LDL between the HZ group and high-fat group after this experiment, indicating that HZ could inhibit the proliferation of vascular smooth muscle cells and reduce the atherosclerotic plaque area and lesion degree of the aorta, coronary artery, and other vessels (Ma et al., 2005).

Experimental studies on hamsters and rabbits with hyperlipidemia showed that polydatin (26) could reduce the serum level of total cholesterol (TC), triglyceride (TG), and low-density lipoprotein cholesterol (LDL-C) levels (Du et al., 2009) (Xing et al., 2009). Zhu found that different doses of polydatin (26) could reduce the level of TC, TG, high-density lipoprotein cholesterol (HDL-C), LDL-C, MDA, and nitric oxide (NO) in hyperlipidemic rats and increase the level of SOD in hyperlipidemic rats, indicating that polydatin (26) could effectively regulate the blood lipid metabolism and correct the disorder of the free-radical metabolism in hyperlipidemic rats so as to exert the effect of anti-AS (Zhu et al., 2006) (Zhu et al., 2005). Similar effects are displayed in rabbits, and administration of polydatin (26) could significantly reduce the rabbit serum levels of TC, TG, and LDL-C in a dose-dependent manner (Xing et al., 2009).

In addition to the effects of the rhizome and roots of HZ on the cardiovascular system, some scholars have researched the leaves of HZ and found that some of the components in the leaves had a hypotensive effect. The anti-hypertensive potency and mechanism of quercetin (48) have been widely reported at home and abroad. Hou found that the renovascular hypertensive rats were fed with normal feeding plus quercetin (48) (30 mg/kg/day) for 8 weeks. Compared with the blank control group, the blood pressure of renovascular hypertensive rats could effectively reduce by adding quercetin (48) to the diet. Second, the concentration of intracellular free calcium in smooth muscle cells of renal artery rings of rats in the quercetin (48) group decreased significantly (Hou et al., 2016).

### 5.2 Effect on the Digestive System

HZ belongs to the liver and gallbladder meridian, which can clear internal and external heat toxins, and has a good therapeutic effect on jaundice in traditional applications (Feng et al., 2008). Modern pharmacology has also found that the pharmacological effects of HZ on the digestive system are mainly focused on the treatment of liver and gallbladder diseases, such as jaundice, hepatitis, liver damage, and so on.

#### 5.2.1 Hepatoprotective Effects

Experiments have proved that Qushi Huayu decoction (QHD) had therapeutic effects on non-alcoholic steatohepatitis (NASH) in mice *in vivo*. The extract of QHD (9.3 g/kg) was to give NASH mice models with liver fibrosis (i.g.) the dose of 10 ml/kg/day for 4 weeks. The results showed that QHD could reduce liver steatosis and inflammation and significantly improve liver fibrosis (*p* < 0.05) (Xin 2019). Another experiment gave NASH model mice QHD (0.93 g/ml) of 10 ml/kg/day for 4 consecutive weeks (i.g.). The results showed that QHD reduced hepatocyte steatosis and inflammation and inhibited intestinal endotoxin leakage in non-alcoholic pancreatitis, and its mechanism was related to downregulating the intestinal MAPK pathway *in vivo* (*p* < 0.05) (Leng et al., 2019).

The resveratrol (25) in HZ also has hepatoprotective effects. Zhou injected resveratrol (25) (10, 20, 30 mg/kg) (i.p.) into a mouse model of CCl4 acute liver injury 24 h before modeling and tested within 24 h after modeling. The results showed that resveratrol (25) (especially the high-dose group 30 mg/kg) inhibited the activation of Nod-like receptor protein 3 (NLRP3) inflammasome, reduced the inflammatory factors interleukin-1β (IL-lβ) and interleukin-18 (IL-18) of liver tissues, and significantly reduced the acute liver injury induced by carbon tetrachloride (*p* < 0.01) (Zhou Yi et al., 2020). Yan proved that emodin (2) had protective effects on acute liver injury induced by lipopolysaccharide in mice. Rats were given emodin (2) (1, 2, 4 mg/ml) 20, 40, and 80 mg/kg/day (i.g.), and the indicators were detected 12 h later. The results showed that emodin (2) could effectively reduce the expression of toll-like receptor 4 (TLR4), tumor necrosis factor-α (TNF-α), and interleukin-6 (IL-6) protein *in vivo*, and it could also reduce the inflammatory response and improve liver injury (*p* < 0.01) (Ding Y. et al., 2018). In addition, experiments have shown that emodin (2) could alleviate CCl4-induced liver fibrosis in mice. The mice were given emodin (2) (20 mg/kg/day) (i.g.) and sacrificed after 48 h. The results indicated that emodin (2) reduced the infiltration of Gr monocytes and inhibited monocyte chemotactic protein 1 (MCP-1) and CCl4 expression and reduced liver inflammation *in vivo* (*p* < 0.01) (Zhao et al., 2018). Li proved that polydatin (26) could improve the diet-induced non-alcoholic steatohepatitis in mice. Mice were injected with polydatin (26) (5 mg/kg) (i.p.) every other day for 4 weeks. The results showed that polydatin (26) could act on the TLR4/nuclear factor protein-kappa B p65 (NF-κB p65) inflammatory signaling pathway, inhibit oxidative stress, and reduce the degeneration and apoptosis of hepatic adipocytes *in vivo* (*p* < 0.01) (Li et al., 2018).

Studies have shown that PCE (polydatin (26), resveratrol (25), and emodin (2)) have a hepatoprotective effect *in vivo*. PCE (80 mg/kg/day, 160 mg/kg/day) was given to fructose-fed metabolic syndrome rats for 11 weeks between 2:30 p.m. and 3:30 p.m. (i.g.). The results showed that it could reduce liver oxidative stress, upregulate peroxisome proliferator-activated receptor-α (PPAR-α) and downregulate sterol-regulatory element binding protein 1 (SREBP1) to restore liver lipid accumulation and liver functions (*p* < 0.05) (Zhao et al., 2019b).

#### 5.2.2 Choleretic Effects

Resveratrol (25) could improve the cholestasis induced by α-naphthyl isothiocyanate (ANIT) in mice. The mice were treated with resveratrol (25) (60 mg/kg) for 3 days and then administered ANIT (60 mg/kg), and blood samples were collected 2 days later. Experiments showed that resveratrol (25) reduced cholestasis in mice and lowered liver inflammation by activating the nuclear receptor farnesoid X receptor (FXR) *in vivo* (*p* < 0.05) (Ding L. et al., 2018). Wu proved that the aqueous extract of HZ could increase the bile secretion of rats. Rats were injected with the extract of HZ (10 ml/kg) into the duodenum, and bile was collected every 1 h within 4 h after the administration. The results showed that the water extract of HZ could increase bilirubin and lower the cholesterol content *in vivo* (*p* < 0.01) (Wu DY. et al., 2014).

#### 5.2.3 Gastrointestinal Protection Effect

PCE (polydatin (26), resveratrol (25), and emodin (2)) have a protective potency on acute gastric injury induced by hydrochloric acid/ethanol. Mice were given 10 ml/kg PCE (100 mg/kg, 300 mg/kg) (i.g.). It was found that PCE (300 mg/kg) could significantly enhance prostaglandin prostaglandin E2 (PGE2), exert anti-oxidant effects, and improve gastric mucosal injury *in vivo* (*p* < 0.0001) (Kim et al., 2020). Another experiment proved that the HZ root extract (resveratrol (25), emodin (2), polydatin (26)) has a synergistic effect to treat ulcerative colitis caused by dextran sulfate sodium, and different doses of the HZ root extract (100, 200, 400 mg/kg) were given for 8 days (i.g.). Experiments had found that high doses (400 mg/kg) improved the anti-oxidant and anti-inflammatory abilities of mice through the NF-κB signaling pathway, and it had an anti-ulcerative colitis effect *in vivo* (*p* < 0.05) (Liu et al., 2018b). PCE also promoted the peristalsis of the large and small intestines of mice with constipation and could excite the isolated intestinal smooth muscle of rabbits, improving the contraction of intestinal muscles of acetylcholine *in vivo* (Men et al., 2009). In addition, polydatin (26) also has a protective effect on the gastrointestinal mucosa of scalded rats (*p* < 0.01) (Yang et al., 2008).

### 5.3 Anti-Tumor Effects

According to clinical observation and traditional Chinese medicine theory, blood stasis is one of the main pathological mechanisms of tumor formation and development. It is recorded in the literature that HZ has the function of promoting blood circulation, dissipating blood stasis, and eliminating symptoms. In modern clinical treatment, HZ has already been confirmed to have anti-tumor activity in many cancers (Tang et al., 2009).

Li studied that HZ decoction, which containing resveratrol (25) and emodin (2) mainly, had inhibitory effects on H22 cells *in vivo*. The hepatocellular carcinoma H22 tumor bearing mice’s anti-tumor rate was 35% (*p* < 0.01) when HZ decoction was given once a day for 15 days (i.g.), 1 ml each time. In addition, a variety of monomer components extracted from HZ also have good anti-cancer effects (Li 2015).

Resveratrol (25) is a hot spot in anti-cancer research. Currently, studies have reported that resveratrol (25) has a good efficacy on a variety of tumors in the digestive system, respiratory system, and reproductive system. In the digestive system, resveratrol (25) induced apoptosis of colon cancer cells at lower concentrations (1 and 10 μmol/L) *in vitro*, which is related to DNA damage and increases in silent information regulator 6 (SIRT6) levels (*p* < 0.05) (San Hipolito-Luengo et al., 2017). Furthermore, resveratrol (25) interrupted the tumor-promoting effect of the tumor microenvironment on colorectal cancer cells by regulating the secretion of paracrine substances and NF-κB signals *in vitro*, and it significantly reduced HCT116 cell survival, migration, and cancer stem cell-mediated metastasis by regulating the resveratrol (25)-Sirt1 axis signal (*p* < 0.05) (Buhrmann et al., 2020). Yang investigated that resveratrol (25) had anti-tumor activity on two gastric cancer cell lines (BGC823 and SGC7901). It inhibited the migration and invasion of human gastric cancer cells by inhibiting metastasis-associated lung adenocarcinoma transcript 1-mediated epithelial to mesenchymal transformation *in vitro* (Yang ZY. et al., 2019). In the respiratory system, by inhibiting protein kinase B/mammalian target of rapamycin (Akt/mTOR) and activating the p38-MAPK pathway, resveratrol (25) inhibited cell proliferation and induced apoptosis and autophagy *in vitro*, which enhances the anti-tumor activity in non-small cell lung cancer (*p* < 0.05) (Wang 2018). Moreover, resveratrol (25) was reported to significantly inhibit the growth of transplanted nasopharyngeal carcinoma of nude mice *in vivo* and *in vitro*. The *in vivo* experiment used a poorly differentiated human nasopharyngeal carcinoma (NPC) cell line CNE-2Z tumor cell mouse model, with resveratrol (25) dissolved in 200 μl of 10% dimethyl sulfoxide in phosphate buffer saline (PBS) and intraperitoneally injected into mice once a day for 3 weeks. The results showed that resveratrol (25) effectively induced apoptosis of nasopharyngeal carcinoma cells, which is related to the regulation of the phosphorylated Akt1/p70 ribosomal S6 protein kinase (pAkt1/p70S6K) signaling pathway (*p* < 0.05) (Zhang MH. et al., 2013). In the reproductive system, by inducing the apoptosis of ovarian carcinoma cells *in vitro*, the mechanism of resveratrol (25) is related to the increase of miR-424-3p level and the decrease of galectin-3 (GAL-3) level (*p* < 0.001) (El-Kott et al., 2019). Zhang confirmed that it induced immunogenic cell death and played an anti-ovarian cancer effect *in vivo* when C57BL/C transplanted tumor model mice were injected intraperitoneally with resveratrol (25) (100 mg/kg) for 21 days (*p* < 0.001). This also provides ideas for the clinical use of resveratrol (25) and immunotherapy in combination (Zhang YK. et al., 2019). Moreover, resveratrol (25) inhibited the proliferation of breast cancer 4T1 cancer cells by inhibiting the cell cycle and inducing apoptosis in a dose- and time-dependent manner *in vitro* (Wu et al., 2019). In another experiment *in vivo*, TC-1 mouse model mice were injected with 10 μl of 1 mmol/L resveratrol (25) for 5 consecutive days. It was reported that resveratrol (25) could downregulate the levels of human papilloma virus (HPV) oncogene E6 and vascular endothelial growth factor (VEGF) tumor protein, showing significant anti-cervical cancer effects (*p* < 0.05) (Chatterjee et al., 2019). In addition, resveratrol (25) also has proved a good therapeutic effect on melanoma (Wu et al., 2017b), glioma cells (Wang H. et al., 2016), leukemia (Luis Espinoza et al., 2013), and so on.

Polydatin (26), another important component of HZ, also has important anti-cancer effects. Polydatin (26) has an effect on hepatocellular carcinoma (HCC). It inhibited the HCC cells through G2/M phase block, suppressed the migration and invasion of HCC cells, and enhanced the apoptosis of HCC cells in a dose-dependent manner *in vitro* (*p* < 0.001) (Jiang et al., 2019). Jin injected polydatin (26) 150 mg/kg into Caco-2 cell model mice for 16 days, which showed that polydatin (26) inhibited the proliferation of colorectal cancer cells and promoted apoptosis by upregulating miR-382 and inhibiting programmed death-ligand 1 (PD-L1) *in vivo* (*p* < 0.01) (Jin et al., 2020). Another *in vivo* study found that 50 mg/kg polydatin (26) was injected into the tumor-bearing mice of human laryngeal cancer Hep-2 cells three times every week for 3 weeks. The outcome showed that polydatin (26) could inhibit cell proliferation and induced apoptosis in laryngeal cancer and HeLa cells via inactivation of the PDGF/Akt signaling pathway (*p* < 0.05) (Li H. et al., 2017). Moreover, polydatin (26) also exerted anti-tumor effects on osteosarcoma cells *in vitro* (Xu et al., 2016).

Other experiments showed that emodin (2) in HZ also had anti-tumor effects. Wang injected azoxymethane (AOM) (10 mg/kg) intraperitoneally on day 0 and then injected dextran sodium sulfate (DSS) (2% w/V) intraperitoneally on day 7 to establish the model of AOM/DSS colitis-related intestinal carcinogenesis. Then, emodin (2) (50 mg/kg) or the vehicle solution (ddH_2_O containing 0.2% tween 80 and 0.5% methylcellulose) was given 2 days before DSS administration for 2, 4, and 14 weeks (i.g.). Studies have shown that emodin (2) inhibited cancer-related intestinal inflammation, reduced the incidence of cancer, and prevented the occurrence of intestinal tumor induced by AOM/DSS *in vivo* (*p* < 0.01) (Zhang YS. et al., 2020). Experiments have also shown that emodin (2) has an inhibitory effect on gynecological cancer cells (cervical cancer-derived cells, Hela; choriocarcinoma-derived cells, JAR; and ovarian cancer-derived cells, HO-8910) *in vitro* (*p* < 0.05). It worked by inducing cell apoptosis and autophagy, blocking the cell cycle, and inhibiting angiogenesis and other mechanisms (Wang Y. et al., 2015). Furthermore, Wang gave hepatocellular carcinoma tumor-bearing mice with anthraglycoside B (6) at the dosage of 80 mg/kg/day (i.g.). The results showed that anthraglycoside B (6) inhibited cell growth and induced apoptosis by regulating Pim family kinases 1 *in vivo* (*p* < 0.01) (Wang et al., 2017). Moreover, anthraglycoside B (6) also has effects on glioblastoma (Li W. et al., 2017) and malignant melanoma (Zhang et al., 2016). Studies have also shown that 2-ethoxystypandrone (24), a compound isolated from the ethyl acetate extract of HZ roots, is a signal transducer and activator of the transcription 3 (STAT3) signal transduction inhibitor, which can strongly block the activation of STAT3 and induced cell apoptosis of HCC cells and HCC cancer stem cells *in vitro* (Li et al., 2019b). Other studies have shown that HZ has not only anti-tumor effects but also has a good therapeutic effect on the multidrug resistance of tumor cells (Eid et al., 2015).

### 5.4 Effect on the Skin

HZ also has the effect of expelling pus and astringing sores. As early as in the “Ben Cao Tu Jing” and other Materia Medical literature studies of past dynasties, there were records about its main treatment of sores, boils, carbuncles, and toxins. Based on the modern study of its astringing sore mechanism, it is found that HZ could restrain scalded wounds and prevent wound infection.

#### 5.4.1 Inhibition of Melanin Production

HZ could inhibit melanin production, and PCE could inhibit tyrosinase, a key enzyme in melanin synthesis. Leu used ethanol to extract four anthraquinones, physcion (1), emodin (2), citreorosein (8), and anthraglycoside B (6), and two stilbenes, resveratrol (25) and polydatin (26). Dose-dependent inhibitory activities (3–30 μmol/L) were observed for the kojic acid treatments. There was a difference in the inhibitory potency on the tyrosinase activity among treatments using the six compounds from HZ. Stilbenes showed no activity on tyrosinase inhibition. On the other hand, moderate to strong inhibitory activity was observed for the anthraquinones. Physcion (1) exhibited the most significant anti-tyrosinase activity compared with the other anthraquinones (*p* < 0.05) (Leu et al., 2008). In order to study the effect of polydatin (26) on melanin production, melan-a melanocytes were treated with 10 μg/ml, 20 μg/ml, and 50 μg/ml polydatin (26). Arbutin, which can inhibit melanin production, was used as a positive control. Polydatin (26) showed significant depigmenting effects in a dose-dependent manner. At the concentrations of 10, 20, and 50 μg/ml, polydatin (26) reduced the melanin content by about 20, 60, and 70%, respectively. These data showed that polydatin (26) had an inhibitory effect on melanin production (Jeong et al., 2010).

#### 5.4.2 Treating Skin Inflammation

HZ could also be used to treat skin inflammation. Edema was induced in both the ears of each mouse by the topical application of 2 µg of 12-O-tetradecanoylphorbol-13-acetate (TPA) dissolved in 20 µl of acetone to both the inner and outer ear surfaces. 30 min after the application of TPA, the inner and outer surfaces of each ear were treated (10 µl to each side) with 50% ethanolic solutions of the PCE in doses of 0.075, 0.15, 0.3, 1.25, and 2.5 mg PCE/ear. Through the above experimental research, Bralley found that the ethanol solution of PCE could inhibit the auricle edema induced by TPA in mice (Bralley et al., 2008). Wang smeared HZ tannin 4 mg to the ears of mice and found that it could significantly inhibit the auricle swelling induced by croton oil. Oral administration of 1 g/kg/day for 4 days could also inhibit this swelling, also indicating that HZ had the effect of anti-skin inflammation (Wang et al., 2004a). Kundu had shown the inhibitory effect of resveratrol (25) on the expression of cyclooxygenase-2 (COX-2) in the mouse skin induced by TPA through experimental studies. Topical application of resveratrol (25) (1 mmol) 30 min prior to TPA led to a statistically significant (*p* < 0.001) decrease in the level of COX-2 protein in the mouse skin 4 h after TPA treatment. Immunohistochemical analysis verified that the TPA-induced expression of COX-2, predominantly localized in the epidermal layer, was significantly (*p* < 0.001) reduced by pretreatment with resveratrol (25). This investigation provided evidence for the potential uses of HZ in cosmeceutical and dermatological products (Kundu et al., 2006).

#### 5.4.3 Treating Skin Burns and Scalds

HZ has been a blind main medicine in traditional Chinese medicine for treating burns and wound healing. We take compound HZ Fengfang Spray (CHFS) as an example. The depilation area of rats was placed on the mouth of a round-bottom flask with side branches (98°C) and fumigated for 13 s to establish a Ⅲ burn model. Then, rats were treated with CHFS. The results showed that CHFS could obviously promote the wound healing of rats from the 21st day, and the wound area of rats decreased faster than that of other groups (*p* < 0.05). The wound healing rate was significantly increased (*p* < 0.05). The hydroxyproline (Hyp) of the CHFS group was different from that of other groups, suggesting that CHFS could promote collagen synthesis and burn surface healing (Zhao et al., 2009). Zhao used the mouse model of deep Ⅱ degree chemical burn induced by sodium hydroxide (NaOH) to observe the effect of HZ ointment on alkali burn wound. The model was made by depilating the back of mice and then saturated with 2 mol/L NaOH solution, which was rubbed on the exposed area of the skin of the back of mice for 30 s to cause Ⅱ degree chemical burn of the skin. The experimental group smeared the wound with HZ ointment. The results showed that HZ ointment could promote epithelial growth, reduce the content of MDA in the wound skin, and shorten the epithelialization time and healing time. This indicated that HZ ointment could obviously promote the healing of alkali burn skin wound in mice (Zhao 2010).

Expansion of blood volume is still the only effective method for early treatment of burns and prevention of shock or further systemic damage in clinical practice. Therefore, actively seeking to effectively control the increase in vascular permeability and reduce post-burn edema and body fluid loss is of great significance for the treatment of burns, especially severe burns. Li burned the depilated area on the back of the rat at 100°C for 30 s by putting in a fresh-keeping bag, causing II to Ⅲ burns. 5 min after the scald, low, middle, and high doses of polydatin (26) (30 mg/kg, 45 mg/kg, 60 mg/kg) were given through the femoral vein for treatment. The experimental results showed that polydatin (26) could inhibit the increase in local vascular permeability of burns and the increase of mesenteric venule vascular permeability after burns. The potency of the polydatin (26) group on mesenteric vascular permeability after burns (*p* < 0.01) was statistically significant compared with the normal saline group. Polydatin (26) treatment could also prolong the survival time of burned rats, and the middle-dose and high-dose groups were significantly different from the normal saline group (*p* < 0.01). By giving polydatin (26) treatment after burns, it could improve the survival time of animals. It suggested that polydatin (26) had a significant therapeutic effect for the treatment of burns (Li 2014).

### 5.5 Antiviral Effects

The characteristics of fever, sweating, high infectivity, and high fatality rate of various acute infectious diseases caused by virus infection are consistent with the “epidemic disease” caused by heat toxin in TCM theory (Liang et al., 2009). It has been shown that most of the clearing heat and detoxification drugs have good anti-viral effects (Jiang et al., 2021). Therefore, HZ, as one of the important representatives of clearing heat and detoxification traditional Chinese medicine, also has obvious antiviral effects.

#### 5.5.1 Anti-Hepatitis B Virus Effects

Jung-San Chang used the HepG2 2.2.15 human hepatoblastoma cell line as the model system, and the HepG2 2.2.15 cells were stably transfected with HBV clone. Then, they added different concentrations of PCE and the water extract of HZ to the culture medium. Their results clearly demonstrated for the first time that PCE could inhibit dose-dependently the production of HBV (*p* < 0.0001) with an effective minimal dosage of 10–2 mg/ml. They also found the possible inhibitive effect of the water extract of HZ in higher doses (3 × 10–2 mg/ml). The expression of the hepatitis B surface antigen (HBsAg) was significantly increased by both PCE and the water extract of HZ dose-dependently (*p* < 0.0001) and time-dependently (*p* < 0.0001) (Chang et al., 2005). Mi studied the anti-HBV drugs of 21 kinds of Chinese herbal medicines such as HZ. Through step-by-step screening *in vitro* and *in vivo*, it was found that PCE (0.18 mg/ml) had a good inhibitory effect on HBV (Mi et al., 1997). Dang found that emodin (2) had a weak and lasting inhibitory effect on HBV replication *in vivo*. The HBV transgenic rats in the experimental group were given normal saline-containing emodin (2) (57.59 mg/kg/day) for 3 weeks. The mice were sacrificed, and serum as well as liver tissues were collected for enzyme-linked immunosorbent assay (ELISA) and histological examination. The results showed that the HBV deoxyribonucleic acid (DNA) of the experimental group was significantly lower than that of the normal group (*p* < 0.05), and the contents of HBsAg, the hepatitis B e antigen (HBeAg), and the hepatitis B core antigen (HBcAg) also decreased (Dang et al., 2009).

#### 5.5.2 Anti-Human Acquired Immunodeficiency Virus Effects

Acquired immuno-deficiency syndrome (AIDS) is an infectious disease caused by HIV, which can seriously destroy the human immune system and severely threaten the life. So far, there is no ideal treatment (Fan et al., 2013). After inoculating sensitive mice with the LP-BM5 virus, it could cause splenomegaly, immunodeficiency, and other similar manifestations of human AIDS, also known as mouse AIDS. This model had been used to screen and evaluate anti-AIDS agents. Jiang infected C57BL/6 mice with the LP-BM5 virus to establish an AIDS model of LP-BM5 virus/C57BL/6 mice. Then, PCE (50 mg/mouse/day, for 4 weeks) was given to the model mice, and the results showed that PCE had anti-viral effects in the mouse model infected with HIV (Jiang et al., 1998). Some scholars had shown that the 70% ethanol extract of HZ had obvious anti-AIDS activity *in vitro*, and its half effective concentration (EC50) was (13.94 ± 3.41) mg/L (Lin et al., 2010).

Yang reported that oral administration of resveratrol (25) (20 mg/kg) could inhibit splenomegaly and the thymus index induced by the Friend Leukemia virus (FLV), which belongs to the same retrovirus as HIV, and significantly increased the level of CD3^+^, CD4^+^, and CD8^+^ lymphocytes in peripheral blood (compared with the model group, *p* < 0.05) (Yang et al., 2006). Raymond had shown that anthraquinones had the effect of anti-HIV, and the IC50 of emodin (2) anti-HIV-1 activity in HZ was 36.3 μmol/L (Schinazi et al., 1990). Through bioactivity-guided fractionation, Lin isolated 20 phenolic compounds from the roots of HZ, and their anti-HIV-1 activities were evaluated. The results showed that the compounds (E)-resveratrol (25), 5,7-dimethoxyphthalide (110), (+)-catechin (57), and anthraglycoside A (5) demonstrated fairly strong anti-viral activity against HIV-1-induced cytopathic effects in C8166 lymphocytes at non-cytotoxic concentrations, with EC50 values of 4.37 ± 1.96 μg/ml, 19.97 ± 5.09 μg/ml, 14.4 ± 1.34 μg/ml, and 11.29 ± 6.26 μg/ml and therapeutic index (TI) values of 8.12, >10.02, >13.89, and >17.71 (Lin et al., 2010). To identify natural products derived from traditional Chinese medicinal herbs that could cause HIV latency reactivation, Wang used the Jurkat T cell line-based J-Lat A2 cells as a model system. They treated J-Lat A2 cells with increasing concentrations of procyanidin C-13,3′,3″-tri-O-gallate (68) (named REJ-C1G3) isolated from HZ and for different time periods. Experiments showed that REJ-C1G3 (68) activates HIV-1 transcription in a dose- and time-dependent manner. Although REJ-C1G3 (68) might not necessarily be the eventual drug used clinically for waking up latent proviruses and curing HIV/AIDS, its identification and characterization in the present study served as an important proof of concept that traditional Chinese medicinal herbs could be a good source for finding such drugs (Wang C. et al., 2015).

#### 5.5.3 Anti-Herpes Simplex Virus Effects

Xue found that HZ decoction (10%) had an inhibitory effect on herpes simplex virus 1 (HSV-1) and herpes simplex virus 2 (HSV-2) by tissue culture of primary monolayer epithelial cells of the human embryonic kidney (Xue 2000). Anthraquinone compounds of HZ were separated by Wang; crystal Ⅰ, crystal Ⅲ, and crystal Ⅳ were obtained. The anti-HSV effects of partial crystal Ⅰ and crystal Ⅳ extracted by ethyl acetate were studied. The results showed that in the Hep-2 cell system, the median effective dose (ED50) of inhibition of proliferation, blocking of infection, and direct killing of HSV-1F strain by crystal Ⅰ were 1.20 μg/ml, 1.86 μg/ml, and 0.97 μg/ml, respectively. The three TI of crystal Ⅰ were 227, 178, and 343. The corresponding values of crystal Ⅳ were 0.09 μg/ml, 2.90 μg/ml, and 0.07 μg/ml, and the three TI were 25,177, 989, and 32,371. Both crystal I and crystal Ⅳ were worthy of development and utilization (Wang et al., 2000). Emodin (2) in HZ had also been found to have anti-HSV effects. In the Hep-2 system, Wang showed that the ED50 of direct killing, proliferation inhibition, and infection blocking of Acyclovir (ACV) to HSV-2 333 strains were 16.45, 10.85, and 18.62 μg/ml, respectively. The ED50 of emodin (2) on direct killing, proliferation inhibition, and infection blocking of HSV-2 333 strains were 1.28, 1.40, and 1.44 μg/ml, respectively, which were smaller than that of ACV, which indicated that emodin (2) had stronger efficacy against HSV-2 333 strains than ACV (Wang 1999).

#### 5.5.4 Anti-Other Virus Effects

In addition to the anti-HBV, HIV, and HSV effects mentioned above, HZ could also be used to fight against other viruses. Yu found that the methanolic extract from HZ (PCME) could effectively inhibit Dengue virus (DENV) infection. They evaluated its anti-DENV effects by immunohistochemical plaque analysis. Briefly, Vero cells were inoculated with DENV-2 in the presence of various concentrations of the test drugs (1, 5 × 10^–3^, 10^–2^, 2 × 10^–2^, 3 × 10^–2^ mg/ml). The experimental results showed that PCME exhibited a dose-dependent effect on the DENV infection, with a concentration up to 3 × 10^–2^ mg/ml completely abrogating the viral infection without inducing significant cytotoxicity (Kuo et al., 2020).

After experimental research, Lin found that HZ and its active components, resveratrol (25) and emodin (2), could attenuate influenza viral replication in A549 cells. Hemagglutinin Type1 and Neuraminidase Type 1 (H1N1) viruses (10 multiplicity of infection (MOI)) were used to infect A549 lung cancer cells for 1 h, and the cells were then treated with a water extract of HZ. The results of the experiment were that HZ inhibited H1N1 replication in A549 cells, and the IC50 value for HZ was 312 μg/ml. Four active ingredients of HZ (rubiadin (19), resveratrol (25), emodin (2), and polydatin (26)) were also tested to determine which compound(s) exhibited inhibitory effects on H1N1 replication in A549 cells. The IC50 values for rubiadin (19), resveratrol (25), emodin (2), and polydatin (26) were >5 × 10^–5^ mol/L, 2.47 × 10^–5^ mol/L, 37.3 × 10^–5^ mol/L, and >5 × 10^–5^ mol/L, respectively. These results indicated that resveratrol (25) and emodin (2) in HZ could inhibit H1N1 virus replication (Lin et al., 2015). The results of Chen also showed that resveratrol (25) significantly inhibited the neuraminidase activity of the H1N1 influenza virus, and the IC50 was 1.298 × 10^–4^ mol/L (Chen et al., 2012).

Wang studied the effects of ribavirin and emodin (2) on Hep-2 cells infected with coxsackievirus B3 (CVB3) and found that the ED50 of direct killing, inhibition of proliferation, and infection blocking of ribavirin were 6.26, 7.55, and 7.50 μg/ml, respectively. The ED50 of emodin (2) in killing CVB3 directly, inhibition of its proliferation, and blocking infection were 1.54, 8.34, and 3.06 μg/ml, respectively, which were lower than that of ribavirin, indicating that emodin (2) had stronger anti-CVB3 effects than ribavirin (Wang 1999). In a model of Xenopus oocytes, Schwarz found that different concentrations of emodin (2) could inhibit the 3a ion channel of the severe acute respiratory syndrome coronavirus (SARSCoV) and human coronavirus OC43 (HCoV-OC43) and inhibit virus release from HCoV-OC43 with a K1/2 value of approximately 20 μmol/L (Schwarz et al., 2011).

In addition, through the analysis of several literature studies on the treatment of virus-related diseases with emodin (2), an active ingredient of traditional Chinese medicine, Yang found that emodin (2) could inhibit the viruses causing respiratory diseases such as SARS, and the gene sequence of SARSCoV-2 is similar to that of SARS-CoV, and they make use of the same cellular receptor Angiotensin converting enzyme 2 (ACE2) to infect cells, suggesting that clinical application of emodin (2) is expected to inhibit the infection and replication of SARS-CoV-2. At the same time, emodin (2)-rich traditional Chinese medicine HZ was extensively used in the treatment of upper respiratory tract infection and viral pneumonia (Liang 2019). Therefore, based on the theory of traditional Chinese medicine, the preclinical study of emodin (2) showed its potential on anti-SARS-CoV-2 (Yang et al., 2020).

### 5.6 Anti-Inflammatory Effects

In TCM, the extract of HZ could dispel wind and relieve pain and benefit joints. It was commonly used in modern clinical treatment of gouty arthritis, rheumatoid arthritis, rheumatoid arthritis, mixed connective tissue disease, and other rheumatic diseases based on the anti-inflammatory and anti-oxidizing effects (Xiao et al., 2018).

HZ has a good effect in treating gouty arthritis (GA). Huzhang Tongfeng granules (HTGs) have anti-inflammatory effects on monosodium urate (MSU)-induced GA model rats. The rats were given HTG 2.14 g/kg/day for 7 days (i.g.). Experiments have shown that the high-dose group (8.56 g/kg) could significantly reduce inflammation (*p* < 0.05). Its mechanism was related to reducing the expression of cysteine-rich 61 (Cyr61) and related inflammatory factors (Zhou et al., 2020c). Furthermore, the Huzhang-Guizhi herb pair (HG) could also be effective for MSU-induced GA *in vivo*. The rats were given HG 9.3 g/kg/day for 7 days (i.g.). The results showed that HG inhibited joint swelling, restrained the expression of inflammatory factors, and had an anti-inflammatory effect (*p* < 0.05) (Han et al., 2016a). Additionally, Jiawei Huzhang San decoction (JHD) could treat autoimmune prostatitis. The chronic non-bacterial prostatitis model rats were given different doses of JHD (i.g.) for 30 days. The medium- and high-dose groups (0.445 and 0.223 g/kg) had good curative effects, and the mechanism of action was related to the regulation of inflammatory factors MCP-1 and GF-BB *in vivo* (*p* < 0.05) (Zhou et al., 2011).

PCE also has anti-inflammatory effects *in vivo*. The dry eye model rats were orally administered with different concentrations of PCE (caftaric acid (77), polydatin (26), and resveratrol (25)) for 5 days. The results showed that the high-concentration group of PCE (250 mg/kg) could inhibit the inflammatory cytokine (IL-6 and TNF-α) expression and activate NF-κB p65 *in vivo* to protect the eyes (*p* < 0.05) (Park et al., 2018). Another study showed that PCE (stilbene and anthraquinone) has a good effect on GA. GA model rats were given different concentrations of PCE (i.g.), once a day for 14 consecutive days, which indicated that high concentrations of PCE (260 mg/kg) exerted anti-inflammatory effects by reducing the levels of uric acid (UA) (*p* < 0.01) and xanthine oxidase (XOD) (*p* < 0.05) (Ren et al., 2016).

In addition, experiments proved that resveratrol (25) and polydatin (26) have anti-inflammatory effects. They could significantly inhibit the production of ROS and NO as well as the level of IL-1β and improve the monocytic cell line (THP-1) inflammation induced by monosodium urate and calcium pyrophosphate *in vitro* (Francesca et al., 2019). Han proved that emodin (2), an important component of HZ, could reduce the activation of NLRP3 inflammasomes, inhibit the secretion of IL-1β, and exert anti-inflammatory effects *in vitro* (Han et al., 2015). Moreover, Qin orally administered different concentrations of anthraglycoside B (6) to a type of II collagen-induced arthritis model rats for 21 days, and he found that it inhibited MH7A in a time- and concentration-dependent manner of cell proliferation and reduced the release of IL-1 and IL-6 and other pro-inflammatory cytokines to exert anti-inflammatory effects *in vivo* (*p* < 0.01) (Geng et al., 2018).

The study also found that polydatin (26) contained in HZ has obvious anti-endotoxin shock effects through anti-inflammatory effects. The researchers injected 0.5% polydatin (26) at a dose of 0.2 ml/kg to healthy and lipopolysaccharide (LPS)-injected rats. Six hours later, an autopsy test found that polydatin (26) upregulates Clara cell secretory protein (CCSP) mRNA expression in rat lungs. Moreover, the CCSP mRNA level in the polydatin (26) pretreatment group was significantly higher (*p* < 0.05) than in the polydatin (26) treatment group. The increase of CCSP expression level is beneficial to reduce lung inflammation, thus playing a role in anti-endotoxic shock (Shu et al., 2011). In addition, emodin (2), another important ingredient in HZ, also has the effect of anti-endotoxic shock. Emodin (2) (0.22 μg/ml) was injected into zebrafish infected with LPS by yolk microinjection. It was found that emodin (2) (0.22 μg/ml) could inhibit the decrease of neutrophils and tissue necrosis in the late stage of infection (24 h). Its mechanism might be related to the inhibition of LPS-activating macrophages and neutrophils to reduce leukocyte production and inflammatory migration and to inhibit the expression of pro-inflammatory factors TNF-α, IL-1β, and IL-6, thus regulating the process of acute inflammation (Zhou et al., 2019).

### 5.7 Anti-oxidize Effects

Choi evaluated the anti-oxidant effect by using HZ 50% ethanol extract (PEE) to scavenge free radicals, total phenol content, and reducing power as indicators. They found that the anti-oxidant activity of PEE showed an increasing trend at different PEE concentrations (50, 100, 200, 400 ug/ml), mainly because of the presence of phenolic compounds (Choi et al., 2020). Lee used the free-radical scavenge examining system, DPPH (2,2-diphenyl-1-(2,4,6-trinitrophenyl)-hydrazyl) assay, and anti-oxidative ferric to investigate the anti-oxidant effect of PCE, which was PCE containing resveratrol (25) mainly. The results showed that the scavenging effect of PCE on the DPPH free radical was dose-dependent, and the concentration of PCE was 20, 50, 100, and 250 μg/ml, respectively. PCE had obvious iron reduction effects at a high dose (250 μg/ml) compared with a low concentration (10 μg/ml) (Lee CC. et al., 2015). Ghanim studied the effects of PCE on oxidative stress and inflammatory stress in normal people. Healthy subjects of a normal weight in the PCE group were given 40 mg of resveratrol (25) a day for 6 weeks. Fasting blood samples were collected before and after treatment. Mononuclear cells were prepared for reactive oxygen species production, RNA extraction, nuclear extraction, and total cell homogenate preparation. The results showed that the PCE group extract could reduce the production of reactive oxygen species in mononuclear cells, suggesting that PCE containing resveratrol (25) could inhibit oxidative stress (Ghanim et al., 2010). Furthermore, Yang made a subacute aging rat model by subcutaneous injection of D-galactose, then gave different doses of resveratrol (25) (30, 60 mg/kg) by continuous gastric perfusion, and then determined various indexes. The results showed that resveratrol (25) could scavenge oxygen free radicals and reduce the contents of SOD and MDA. The results indicated that resveratrol (25) had strong scavenging effects on free radicals and anti-lipid peroxidation (Yang et al., 2013).

However, Su used human umbilical vein endothelial cells (HUVECs) and the human breast cancer MDA-MB-231 cell line as research objects. The anti-oxidative effects of resveratrol (25) and polydatin (26) were evaluated by the phenanthroline-Fe_2_^+^ method and the hydrogen peroxide (H_2_O_2_)-induced oxidative injury cell model *in vitro*. It was found that the scavenging activity of polydatin (26) on the hydroxyl radical (·OH) was higher than that of resveratrol (25), and their scavenging ability of OH at low concentrations was higher than that of vitamin C (VC). Resveratrol (25) could significantly reduce the oxidative damage and improve the viability of HUVEC and MDA-MB-231 cells in the concentration range of 10–50 mmol/L, but its protective effect was weakened in 100 mmol/L. The protective effect of polydatin (26) on HUVEC cells was similar to that of resveratrol (25). The results showed that resveratrol (25) had obvious protective effects on H_2_O_2_-induced cell injury, and the scavenging activity of polydatin (26) on OH *in vitro* was higher than that of resveratrol (25) (Su et al., 2013). Jin established a method for the detection of superoxide radicals (O_2_^−^), OH, and H_2_O_2_ produced by acellular systems. The results showed that polydatin (26) could scavenge these oxygen free radicals and H_2_O_2_ in a dose-dependent manner, and the IC50 were 14.6 μmol/L, 29.6 μmol/L, and 13.0 μmol/L, respectively, indicating that polydatin (26) was a free-radical scavenger. It had obvious anti-peroxidation effects (Jin et al., 1993). He found that polydatin (26) could reduce ultraviolet (UV) B radiation-induced HaCaT cell death in a dose-dependent manner (20, 40, 80 μg/ml). After treatment with polydatin (26), ROS production induced by UVB radiation decreased in a dose-dependent manner (20, 40, 80 μg/ml) (He et al., 2012). In order to research the protective potency of polydatin (26) on oxidative stress injury (OSI) of HUVECs induced by H_2_O_2_, Qiao placed HUVECs at different concentrations (0.1–10 μg/ml) of polydatin (26) or different concentrations of H_2_O_2_ (400 μmol/L) and incubated for 4 h. After H_2_O_2_ treatment, the cell survival rate decreased to 48%. There was no significant difference between the H_2_O_2_ group and the low-dose polydatin (26) group (0.1 μg/ml), but when the concentration of polydatin (26) was 3 μg/ml or higher, the cell survival rate increased to 72%. On this basis, 400 μmol/L H_2_O_2_ and 3 μg/ml polydatin (26) were selected for further experiments. It was found that the morphological changes of cells and the distance between scratches were increased after H_2_O_2_ treatment. Compared with the H_2_O_2_ group, the cell adhesion rate of polydatin (26) treatment group was significantly increased and the scratch spacing was shortened by nearly 40%. In addition, the polydatin (26) group not only induced a remarkable decrease in the level of lactate dehydrogenase (LDH) by more than 50% and ROS by 18% but also significantly attenuated the changes in the content of GSH-Px by 33% and SOD by 60%. The experimental results indicated that polydatin (26) had a protective effect, and its mechanism might be connected with the protein kinase c (PKC) pathway (Qiao et al., 2016). Liang established a rat cerebral I/R model by separating and ligating bilateral common carotid arteries and vagus nerves for 1.5 h and then loosening and allowing blood flow to reflux. The anti-free radical effects of polydatin (26) (6, 12, 18 mg/kg) were observed. The results showed that polydatin (26) could reduce the content of lipid peroxides and increase the activities of SOD, catalase (CAT), and glutathione peroxidase (GSH-Px) in brain tissues in various degrees, and the effect was significantly related to the dose. Polydatin (26) 12 mg/kg had the best effect when injected intravenously (Liang et al., 1996).

Moreover, the experiments of Fu also showed that HZ tannin 2.5 μg/ml significantly inhibited the O_2_^−^, 60 μg/ml significantly antagonized H_2_O_2_-induced blood, and 160 μg/ml had obvious scavenging effects on OH (Fu et al., 1994).

### 5.8 Anti-Pulmonary Fibrosis Effects

HZ has the effect of inhibiting PF in a variety of animal models. Liu proved that Yangfei Huoxue decoction (YHD) reduced bleomycin-induced PF in rats by inhibiting the level of vascular endothelial growth factor and inflammatory factor interleukin 1β (IL-1β). It has shown that mice were injected intratracheally with bleomycin and given different concentrations of YHD. After 7, 14, and 28 days of administration, blood was collected and the results showed that YHD had lower IL-1β values in the middle (9.18 g/kg/day) and low (4.59 g/kg/day) concentration groups, which were better than those of the control group *in vivo* (*p* < 0.01) (Liu et al., 2019). In the same animal model mentioned above, Chen researched that YHD inhibited PF by regulating the immune system, and its molecular mechanism might be connected with the modulation of the Notch signaling pathway (*p* < 0.05) (Chen H. et al., 2020).

In addition, other active ingredients in HZ also have a therapeutic effect on PF. Wang established a rat model of PF induced by bleomycin and given different concentrations of resveratrol (25) and dexamethasone (i.g.) and finally found that on the 7th day, the high-dose resveratrol (25) group (100 mg/kg) effectively inhibited the expression of hypoxia inducible factor-1α (HIF-1α) and NF-κB to inhibit PF (*p* < 0.05) (Wang ZY. et al., 2021). Furthermore, it was also reported that the fine particulate matter (PM2.5) mice model of 5 months was given 0.1 ml resveratrol (25) (50 and 100 mg/kg/BW) every 2 days (i.g.), and the results showed that resveratrol (25) could reduce PF and related inflammation by inhibiting the activation of autophagy-related NLRP3 inflammasome *in vivo* (*p* < 0.01) (Ding et al., 2019). In *in vitro* experiments, emodin (2) significantly inhibited the activity of neutrophil elastase in rat alveolar type II epithelial cell line RLE-6TN and human alveolar epithelial cell line A549 through the Notch1 signal, thereby inhibiting the mesenchymal transition of alveolar epithelial cells and reducing the occurrence of PF (*p* < 0.01) (Zhou LS. et al., 2020). Not only that, emodin (2) and polydatin (26) could also alleviate bleomycin-induced PF (Tian et al., 2018; Liu et al., 2020).

### 5.9 Protective Effect on Ischemia/Reperfusion Injury

I/R injury refers to the phenomenon that tissue damage is aggravated and even irreversible damage occurs when blood flow is restored on the basis of tissue ischemia (Papadopoulos et al., 2013). HZ and a variety of active ingredients could ameliorate the I/R injury caused by different conditions.

#### 5.9.1 Ameliorating Cerebral Ischemia/Reperfusion Injury

The prescription Tongfu Huoxue decoction containing HZ had been proved to have a protective effect on the brain tissue of rats with intracerebral hemorrhage. Liu made the model by drilling holes in the surface of rat skulls and injecting normal saline 1.2 μl containing collagenase with a syringe. After modeling, Tongfu Huoxue decoction was intragastrically infused twice a day, each time 3 ml. The results showed that compared with the model group, the mortality rate and the improvement of hemiplegia in the treatment group were significantly lower than those in the model group (*p* < 0.01). There were also significant differences in reducing MDA content and increasing NO content and SOD activity (*p* < 0.05), indicating that Tongfu Huoxue decoction had a good therapeutic effect on cerebral hemorrhage (Liu et al., 2006).

Guo observed that polydatin (26) injection (7.5, 15, 30 mg/kg) could significantly ameliorate brain edema, reduce the formation of lipid peroxidation, reduce the accumulation of lactic acid, and inhibit monoamine oxidase. The intensity of action was related to the dose and had protective effects on acute global cerebral I/R injury in rats (Guo et al., 2005). In the present study, Gao evaluated the neuroprotective effect of polydatin (26) in preventing apoptosis following induction of focal cerebral ischemia by middle cerebral artery occlusion (MCAO) in rats. Polydatin (26) (30 mg/kg) was administered by caudal vein injection 10 min prior to I/R injury. The modeling method was as follows: a nylon monofilament was inserted proximally into the internal carotid artery through the external carotid artery and pushed forward until a slight resistance was felt, which indicated occlusion of the origin of the middle cerebral artery (MCA). The nylon monofilament remained in place for 2 h and then retracted, allowing reperfusion of the ischemic region. 24 h following I/R injury, ameliorated modified neurological severity scores (mNSSs) and a reduced infarct volume (*p* < 0.01) were observed in the polydatin (26)-treated group. Moreover, treatment with polydatin (26) decreased cell apoptosis compared with the other group (*p* < 0.01) (Gao et al., 2016).

Wang made the model of focal cerebral ischemia in rats by Nagasawa H’s improved method and observed the therapeutic effect of 20 mg/kg resveratrol (25) on the model animals. The results showed that resveratrol (25) could improve the neurofunctional score of rats and reduce the area of cerebral infarction (*p* < 0.05). At the same time, resveratrol (25) could increase the activity of SOD in brain lysate and decrease the contents of MDA and myeloperoxidase (MPO). The experimental results indicated that resveratrol (25) had a therapeutic effect on focal cerebral I/R injury in rats by increasing the clearance rate of free radicals (Wang SQ. et al., 2012).

Leung ligated the right MCA of the rat with a 10-O suture. After 60 min of ischemia, the ligation was removed to generate reperfusion injury. Then, rats in the administration group were intraperitoneally injected with emodin (2) 15 mg/kg. The result of the experiment was that emodin (2) reduced the infarct volume and cell death following focal cerebral I/R injury. Moreover, emodin (2) treatment reduced reactive oxygen species (ROS) production and glutamate release under conditions of ischemia/hypoxia (Leung et al., 2020).

#### 5.9.2 Ameliorating Renal Ischemia/Reperfusion Injury

Meng used oxygen-glucose deprivation (OGD), followed by reoxygenation (OGD/R) to treat primary renal tubular epithelial cells (RTECs) to simulate the *in vitro* I/R injury model. For the evaluation of secretion of sonic hedgehog (Shh) in RTECs treated with polydatin (26) under OGD/R conditions, 10, 20, and 40 μmol/L of polydatin (26) were added into the cell culture medium, respectively. Furthermore, the models of renal I/R injury were established in mice by the clamping unilateral (left) renal pedicles for 30 min with non-traumatic microaneurysm clamps, followed by clamp release to allow reperfusion. Then, the mice were intraperitoneally injected with polydatin (26) (40 mg/kg). On the 3rd day after modeling, the animals were sacrificed, and the kidney tissue was taken for further analysis. Their results showed that I/R injury induced the secretion Shh, upregulated Patched and Smoothened, and enhanced the nuclear translocation and target gene transcription of Glioblastoma 1 in renal I/R injury models, which were further upregulated after the administration of polydatin (26) significantly and in turn exerted prominent nephroprotective effects against cell apoptosis and oxidative stress (Meng et al., 2016). Li simulated the process of renal I/R injury by using the hypoxia-reoxygenation method in normal rat kidney cell-52E (NRK-52E) cells cultured *in vitro*. The cells were treated with polydatin (26) at different concentrations (20, 40 mg/L). The results showed that polydatin (26) downregulated the mRNA and protein expression of TLR4 in a concentration-dependent manner and decreased the protein expression of NF-κB, the downstream signal molecule of TLR4, and the protein expression of inflammatory cytokines TNF-α and IL-1β (*p* < 0.05) (Li Y. et al., 2014).

#### 5.9.3 Ameliorating Pulmonary Ischemia/Reperfusion Injury

Wang injected the heparin (1 mg/kg) anti-coagulant intravenously, and the rabbit pulmonary I/R injury model was established according to the Sekido method, which was blocking the left hilum to stop the blood supply and ventilation to cause left lung ischemia and loosening the blocking band to restore the blood supply and ventilation to form reperfusion after reaching the predetermined time. After intravenous injection of 0.2% polydatin (26) solution according to 2.5 mg/kg, it was observed that the content of MDA in the polydatin (26) group was lower than that in the I/R group, and the activity of SOD was significantly increased (*p* < 0.01), suggesting the protective effect of polydatin (26) on pulmonary I/R injury (Wang et al., 2008). Jin also replicated the rabbit pulmonary I/R injury model according to the Sekido method. 0.2% polydatin (26) solution was injected intravenously with 2.5 mg/kg immediately before ischemia and reperfusion. It was found that the lung tissue injury in the polydatin (26) group was significantly less than that in the model group, and the expression of TLR4, NF-κB p65, and intercellular adhension molecule-1 (ICAM-1) mRNA was significantly lower than that in the I/R group. This suggested that polydatin (26) might reduce the inflammatory reaction and pathological injury caused by lung I/R injury by regulating the TLR4 pathway (Jin et al., 2009).

In addition, other studies had shown that polydatin (26) could reduce hepatic ischemia-reperfusion (HIR) injury in rats. Xu injected 10 and 40 mg/kg polydatin (26) or combined with the Nrf2 inhibitor into the treatment group for 3 consecutive days before modeling. The model was established by anesthetizing SD rats and then clamping the left portal vein branch of the liver with blood vessels to cause 70% hepatic ischemia. After 45 min ischemia, the clamps were removed and reperfused for 6 h till the reperfusion was completed. The aortic blood samples were collected, and the rats were killed to remove part of the liver tissue for follow-up experiments. The results showed that the serum ALT and AST activity, pathological score, TNF-α, IL-1β, IL-6, MDA activity in liver tissues, and hepatocyte apoptosis rate decreased significantly in the high-dose polydatin (26) group (*p* > 0.01). On the contrary, the corresponding indexes in the model group and high-dose polydatin (26) combined with the Nrf2 group were significantly increased (*p* > 0.01). The results proved that polydatin (26) might improve HIR injury in rats by activating the Nrf2/HO-1 signal pathway and inhibiting HIR-induced inflammation, oxidative stress, and hepatocyte apoptosis (Xu et al., 2021).

### 5.10 Anti-Nerve Injury Effects

#### 5.10.1 Anti-Depressant Effects

Depression is a neuropsychiatric disorder with persistent depression and decreased interest. Relevant experiments have proved that PCE has a good effect in the treatment of depression. Wang respectively administered the mouse water extract of HZ (1.5, 3 g/kg/day) and the alcohol extract of HZ (HZ-E) (1.5, 3 g/kg/day) for 7 consecutive days (i.g.) and then carried out the tail suspension test (TST), forced swim test (FST), and opening field test (OFT). The results showed that with OFT to eliminate false positive results, both the water extract of HZ and HZ-E could shorten the immobility time of TST and FST and show obvious antidepressant activity *in vivo*, and the effect of 3 g/kg water extract of HZ was better (*p* < 0.01) (Wang et al., 2013).

Many studies have proved that resveratrol (25) has a better effect on depression. Zhu gave chronic stress rats 10 ml/kg different concentrations of resveratrol (25) (2.5, 5, 10 mg/kg) (i.g.) for 12 days and conducted behavioral tests at different times, including TST, FST, the elevated plus-maze test (EPM), and the hole-board test (HBT). The results showed that after 14 days of treatment with resveratrol (25), 10 mg/kg resveratrol (25) could significantly reduce the TST and FST immobility time and increase the percentage of rats with open arms and the time to open arms as well as the time for EPM and HBT to probe acupoints (*p* < 0.05). It proved that resveratrol (25) could inhibit phosphodiesterase 4D (PDE4D) and activate the cyclic adenosine monophosphate/protein kinase A/phosphorylated vasodilator-stimulated phosphoprotein/phosphorylated camp response element binding protein/brain derived neurotrophic factor (cAMP/PKA/pVASP/pCREB/BDNF) signaling pathway to improve depression *in vivo*, and it showed a dose dependence (Zhu et al., 2019). In another experiment, chronic unpredictable mild stress (CUMS) mice were given 80 mg/kg/day resveratrol (25) (i.g.) for 4 weeks. The results showed that the mechanism of resveratrol (25) in reversing CUMS-induced abnormal behavior is related to anti-oxidant effects; resveratrol (25) could also play an anti-depressant role by upregulating the levels of phosphor-Akt and mTOR in the hippocampus and prefrontal cortex (PFC) *in vivo* (*p* < 0.05) (Liu et al., 2016). In addition, resveratrol (25) also has anti-depressant effects on mice with depression induced by chronic restraint stress. Its mechanism of action was achieved by inducing cell apoptosis and upregulating the levels of BDNF and the phosphorylated form of extracellular signal-regulated protein kinase (pERK) *in vivo* (Wang XE. et al., 2016).

#### 5.10.2 Improve Alzheimer’s Disease

AD is a degenerative disease of the central nervous system that occurs in the elderly. It is often clinically manifested as symptoms of general dementia such as memory impairment and behavior changes. Studies have shown that both the water extract of HZ and HZ-E have the effect of improving AD *in vivo*, and the effect of HZ-E is better. The AD mouse model was established by using β-amyloid fragments. After the Morris water maze experiment, 10 ml/kg of the water extract of HZ and HZ-E were given on the 6th day (i.g.), once a day for 30 consecutive days. The water maze test found that the incubation period of mice in the HZ-E group was significantly shortened (*p* < 0.05). HZ-E played an anti-AD role through anti-oxidation, reducing the production of inflammatory mediators and regulating the cholinergic system (Zhu et al., 2014). As for another experiment, AD model mice were given different doses of HZ-E (20 ml/kg) (i.g.). After 21 days, the learning and memory abilities of mice were tested by Y maze and Morris water maze experiments. Yin proved that HZ-E (18 g/kg) could improve AD mice’s learning and memory ability and reduce the expression of tau protein phosphorylation *in vivo* (*p* < 0.01) (Yin et al., 2018). Under the above experimental model, Zhang proved that the mechanism of improving AD was related to the adenosine-monophosphate-activated protein kinase/peroxisome proliferator-activated receptor gamma costimulator 1-α/BDNF/tyrosine receptor kinase B (AMPK/PGC-1α/BDNF/TRKB) signaling pathway *in vivo* (*p* < 0.05) (Zhang EF. et al., 2019).

In addition, Li gave APP/PS1 double-transgenic mice emodin (2) (10 mg/kg/day, 20 mg/kg/day) (i.g.) for 8 weeks. After behavioral testing, it was found that emodin (2) had anti-oxidant activity; it could improve memory and learning ability and reduce anxiety (*p* < 0.001) *in vivo*. Therefore, it was expected to be a drug for the treatment of AD (Li et al., 2021). Apart from this, polydatin (26), the monomer of HZ, could act on AD model cells, promote autophagy, reduce oxidative stress, and improve mitochondrial dysfunction to play a neuroprotective role *in vitro* (Wang 2018). HZ also has a good effect in the treatment of neurodegenerative diseases such as Parkinson’s disease (PD) and aging-related nerve damage diseases. Lipopolysaccharide (LPS)-induced PD model rats were given polydatin (26) (25, 50, 100 mg/kg) (i.g.) for 4 consecutive weeks. The results showed that polydatin (26) could inhibit the activation of microglia and the release of pro-inflammatory mediators in a concentration-dependent manner *in vivo*, which improves the motor dysfunction (*p* < 0.01) (Huang et al., 2018).

Rats of different ages were fed 0.72 mg/day of resveratrol (25)-supplemented diet (120 mg/kg) for 8 weeks and then subjected to behavioral testing. The results showed that resveratrol (25) could reduce age-related motor nerve decline by promoting the survival of dopamine neurons and activating the extracellular regulatory protein kinase-1/2 (ERK1/2) pathway (*p* < 0.05) (Allen et al., 2018).

### 5.11 Effect on the Endocrine System

#### 5.11.1 Metabolic Regulation Effects

HZ has the function of metabolic regulation; the animal experiments showed that PCE could be used to treat metabolic syndrome and regulate blood glucose and lipid metabolism. Aleksandar fed the rats with standard pellet feed and cholesterol to induce hyperlipidemia and added 10% fructose and streptozotocin to the drinking water to induce type 2 diabetes (T2DM). The results showed that the total TG concentration (*p* < 0.05) and LDL-C concentration (*p* < 0.05) between the two groups had a statistically significant decrease, while the HDL-C content increased significantly (*p* < 0.05). Gavaged with aqueous resveratrol (25) aqueous solution (20 mg/kg), the results showed that the TG concentration (*p* < 0.05) and HDL-C concentration (*p* < 0.05) of rats were significantly reduced, both of which were statistically significant. The content of HDL-C increased significantly (*p* < 0.05). The levels of creatinine (*p* < 0.05) and uric acid (*p* < 0.05) were also significantly reduced (Raskovic et al., 2019). Li showed that compound PCE had a certain hypolipidemic effect. PCE of 4, 8, and 12 g/kg could improve the blood lipid level of hyperlipidemia rats induced by high-fat diet, and the potency of the high-dose group and middle-dose group was better than that of the low-dose group (Li B. et al., 2014). Sohn took male SD rats as the research object and induced diabetes in rats by intraperitoneal injection of 60 mg/kg of streptozotocin (STZ). The ethanol extract of HZ (100 mg/kg and 350 mg/kg) was administered to STZ-induced diabetic rats daily. The study showed that preparation of PCE treatment ameliorated the enhanced diabetes-induced renal dysfunction, such as albuminuria glomerular matrix expansion. In this study, they also found that the treatment of diabetic rats with PCE ameliorated mesangial expansion by inhibiting the binding activity of platelet-derived growth factor-BB (PDGF-BB) to its receptor, PDGFR-β (Sohn et al., 2014). Oral gavage PCE was performed in the treatment group at the level of 0.2 g/kg of body weight. Sheng found that PCE could reduce the blood sugar of diabetic rats and make lipid indexes such as LDL-C, HDL-C, and TG tend to be normal (Sheng et al., 2019).

In addition to PCE, polydatin (26) could partially restore glucose and lipid metabolism in high-fat and high-sugar diabetic rats. Orally administered polydatin (26) taken for 8 weeks at 75 mg/kg reduced the levels of fasting blood glucose, glycosylated hemoglobin glucose (HbA1c), glycosylated serum protein, TC, TG, and LDH-C in diabetic rats (Şöhretoğlu et al., 2018). Wang demonstrated that long-term emodin (2) (3 μmol/L) administration improved glucose tolerance and ameliorated other metabolic disorders in ob/ob mice by the inhibition of 11β-hydroxysteroid dehydrogenase 1 (11β-HSD1) activity in adipose tissues (Wang YJ. et al., 2012). Emodin (2) (40 mg/kg/day) effectively improved renal dysfunction in diabetic nephropathy (DN) rats possibly through its inhibition of the activation of the p38 mitogen activated protein kinase (MAPK) pathway and downregulation of the expression of fibronectin (Wang et al., 2006). Zhao established the rat model of hyperlipidemia by feeding high-fat diet, which was treated with polydatin (26) 100 mg/kg/day. The results showed that polydatin (26) could reduce the levels of TC, TG, and LDL-C and oxidized low-density lipoprotein (ox-LDL) in serum of hyperlipidemic rats, increase the level of HDL-C, and decrease the ratio of LDL-C/HDL-C (Zhao et al., 2019a). Xing used a high-fat/cholesterol diet to feed male Japanese giant-eared rabbits for 3 weeks to create a hyperlipidemia model. The animals were given polydatin (26) (25, 50, 100 mg/kg/day) through tracheal intubation. The results of blood lipid determination were that except for HDL-C, TC, TG, and LDL-C were significantly lower than those of the control group; the difference was statistically significant (*p* < 0.05), and the TC/HDL ratio was significantly reduced (Xing et al., 2009).

Quercetin (48) and quercitrin (47) in the leaves of HZ also had the effect of lowering blood lipid and blood sugar. Yan used diabetic Goto-Kakizaki (GK) rats as models and administered low and high doses of quercetin (48) (50 mg/kg, 100 mg/kg) daily to rats to study the liver function and blood lipid levels of quercetin (48) diabetic GK rat influences. The results showed that quercetin (48) could reduce rat serum alanine aminotransferase (ALT), aspartate aminotransferase (AST), TG, TC, and LDH-C levels and could protect the liver of diabetic GK rats and regulate blood lipids (Yan et al., 2018). Xing used high-fat feed to feed rats to form a hyperlipidemia model and gavage rats with low, medium, and high doses of quercitrin (47) (10 mg/kg, 20 mg/kg, 40 mg/kg). The results showed that high-dose quercitrin (47) could significantly reduce the levels of TC, TG, and LDL-C and increase the level of HDL-C in hyperlipidemic rats, which indicated that quercitrin (47) could regulate blood lipid (Xing et al., 2014).

#### 5.11.2 Estrogenic Effects

Some components extracted from HZ have estrogenic effects. The separation of emodin (2) and anthraglycoside A (5) from the methanol extract of HZ could enhance the proliferation of Michigan Cancer Foundation-7 (MCF-7) cells, which was sensitive to estrogen. The ethyl acetate fragment (Hzs1and Hzs6) in the ethyl acetate extract of HZ showed strong estrogenic activity (Matsuda et al., 2001).

## 6 Clinical Uses

### 6.1 External Use

The external use of HZ is mainly for the treatment of skin diseases, burns, scalds, snake and scorpion bites, bone injuries, and other diseases, and the effect is very significant. There are many dosage forms commonly used in clinical practice, including tinctures, ointments, sprays, decoctions, and so on.

HZ has a long history of being used to treat burns. According to reports, 120 cases of burn patients were treated with “Compound HZ Tincture” (Song et al., 2002). After spraying every 2–4 h, 115 cases (95.8%) were effective and 5 cases (4.2%) were ineffective. Yan used “Compound Huqing Spray” to treat patients with II-degree facial burn, which was administered once every 4 h. The results showed that the pain was rapidly relieved after the administration of the drug, and the facial swelling was reduced after 48 h, earlier than 72 h in the positive drug group. In addition, the positive rate of bacteria in the “Compound Huqing Spray” treatment group was 12.6%, which was significantly lower than that in the positive drug group (67.6%) (*p* < 0.01), which could shorten the wound healing time (“Compound Huqing Spray” treatment group: 9.4 ± 2.1d, positive drug group (Yang 2010): 12.4 ± 1.8d, *p* < 0.05). In another research, nine patients with depth II-degree burn were treated with “Bai Ji-HZ Glue”. They were treated with this medicine two to three times a day. After 20 days, all patients healed without infection. At the same time, this glue was also used to treat three children with superficial II-degree scald (Xia 1999). The medicine was applied one to two times a day, and the patients healed in 10 days. Moreover, preparations mainly containing HZ, such as “Lv Zi Cream” (Song et al., 1992), “Bao Hu Cao Ointment” (Zhang et al., 2000), “Compound HZ Film” (Xu et al., 1998), and “HZ Paste” (Huang et al., 2005), are very effective in the treatment of burns. Both of them can promote wound healing, without any scars, and are easy to make with low cost (Table 4).

**TABLE 4:** Representative preparations of HZ in treatment of burns.

Name	Type	Main Herbs	Clinical Research	References
Compound HZ	Tincture	HZ, Phellodendri Chinensis Cortex, Sanguisorbae Radix, Ulmi pumilae Cortex	Number of patients: 120; recovery rate: 95.8%; recovery cycle: 14.67 d	Song et al. (2002)
Compound Huqing	Spray	HZ, Astragali Radix, Bletillae Rhizoma, Lonicerae Japonicae Flos, Phellodendri Chinensis Cortex, Sophorae Flavescentis Radix, Arnebiae Radix, Salviae MiltiorrhizaeRadix et Rhizoma	Number of patients: 50; recovery rate: 100%; recovery cycle: 9.4 d	Yang (2010)
Bai Ji-HZ	Glue	HZ, Bletillae Rhizoma	Number of patients: 11; recovery rate: 100%; recovery cycle: 7–20 d	Xia (1999)
Lv Zi	Cream	HZ, Arnebiae Radix, Chloramphenicol	Number of patients: 100; recovery rate: 100%; recovery cycle: 2–5 d	Song et al. (1992)
Bao Hu Cao	Ointment	HZ, Arnebiae Radix, Bletillae Rhizoma	Number of patients: 41; recovery rate: 100%; recovery cycle: 5–12 d	Zhang et al. (2000)
HZ	Paste	HZ, Borneolum Syntheticum	Number of patients: 250; recovery rate: 93.6%; recovery cycle: 5–12 d	Huang et al. (2005)
Compound HZ	Film	HZ, Phellodendri Chinensis Cortex, Sanguisorbae Radix, Bletillae Rhizoma	Number of patients: 69; recovery rate: 100%; recovery cycle: 2–7 d shorter than the positive drug group	Xu et al. (1998)

Resveratrol (25) was extracted from HZ and prepared into spray (resveratrol (25) 0.05% and carboximethyl-β-glucan 0.33%). Seventy-six children’s patients were instructed to apply two sprays (100 μL/spray) for nostril 3 times/day. After 2 months, resveratrol (25) plus carboximethyl-β-glucan treatment significantly reduced (Miraglia Del Giudice et al., 2014) cough severity, during both the day (*p* ≤ 0.001) and the night (*p* ≤ 0.0001), wheezing intensity (*p* < 0.01), and short acting β2-agonist use (*p* < 0.01). In another report, 128 cases of herpes zoster were treated with the liniment containing HZ and Calamina. The total effective rate was 96.88%, and the cure rate of mild patients (98.55%) was higher than that of severe patients (94.51%) (Wang ZQ. et al., 2010). The roots of HZ were decocted and filtered to treat patients with colpomycosis (Li 1986). The patients sit in the bath for 10–15 min a day, and the cure rate was 100%. Zhang took HZ, Phellodendri Chinensis Cortex, Rhei Radix et Rhizoma, and other herbs into suppository to treat 52 patients with chronic prostatitis. The total effective rate is 80.8%, which is higher than the 65.0% of the control group (Zhang ZR. et al., 2008). The suppository is easy to use and non-irritating. Besides, the anti-inflammatory and analgesic tincture containing HZ developed by Zhang can significantly reduce swelling and relieve pain (Zhang et al., 1997). Clinically, 130 cases of patients with tennis elbow, acute lumbar sprain, and chronic lumbar muscle strain were treated, with a total effective rate of 93.8%. Li prepared emplastrum containing HZ for the treatment of protrusion of the lumbar intervertebral disc. The results proved that the overall potency of the treatment group (92.45%) was better than that of the control group (73.58%) [95% CI of the control group was (0.518, 0.655), R = 0.587; 95% CI of the treatment group was (0.324, 0.463), R = 0.413] (Li 2019).

### 6.2 Internal Use

200 mg of the HZ extract standardized to contain 20% trans-resveratrol was given to healthy male professional basketball players. After 6 weeks of supplementation, there was a significant reduction in plasma levels of TNF-a and IL-6. This indicated that 6 weeks of PCE containing resveratrol supplementation could reduce the inflammation in male professional basketball players (Zahedi et al., 2013). In addition, resveratrol (25) significantly improved the fasting plasma glucose (−0.29 mmol/L, 95% CI: −0.51, −0.06, *p* < 0.01) and insulin levels (−0.64 U/mL, 95% CI: −0.95, −0.32, *p* < 0.0001) (Zhu et al., 2017). The drug also reduced the homeostasis model assessment of insulin resistance (HOMA-IR) index, systolic blood pressure, and diastolic blood pressure among participants with type 2 diabetes mellitus (T2DM).

HZ is also often prepared in a variety of oral dosage forms, such as liquid, pill, and granules. For instance, Li Dan Pai Shi Pill was used to treat 110 patients with stones. The shortest duration of medication was 20 d, while the longest duration was 72 d (Chen 2007). No adverse reaction occurred in any patient, and the total effective rate was 85.45%. Compound HZ Yi Gan granules were prescribed for the treatment of chronic hepatitis B to 43 patients for 6 months, and the total effective rate was 81.40% (95% CI = 69.77–93.03%), which was higher than that of the control group [62.79%, (95% CI = 48.34–77.24%)] (Han 2014). Fu used single HZ to make a kind of oral liquid to treat 160 cases of acute upper gastrointestinal bleeding (Fu et al., 2006). The total effective rate was 96.87%, which was higher than 87.91% in the control group (*p* < 0.01). Additionally, HZ has great benefits for gout sufferers. Yang treated 1,000 gout patients with the Compound HZ mixture (Yang 2019). After treatment (3 times/d for 8 weeks), the levels of serum uric acid, total cholesterol (TC), triglyceride (TG), and low-density lipoprotein-cholesterol (LDL-C) were decreased, and the high-density lipoprotein-cholesterol (HDL-C) level was increased (*p* < 0.05). To accomplish the clinical evaluation of HZ Tongfeng granules in the treatment of acute gouty arthritis, Zhou administrated 64 gout suffers with HZ Tongfeng granules twice a day, one pack (12 g) each time, and took 7d as a course of the treatment. After treatment, the blood uric acid (BUA), serum creatinine (Scr), and white blood cell count (WBC) in the treatment group decreased (*p* < 0.05, *p* < 0.01) (Zhou et al., 2020b). The treatment group showed an effective rate of 82.8%, and the total effective rate is 98.4%.

### 6.3 Other Applications

Lee dealt with possibilities of transferring HZ into a cosmetic cleanser. Healthy volunteers (n = 23) aged 20–50 years were asked to apply the test cleanser which contains HZ to the face. Then, the oil content decreased by 77.3%, from 6.19 to 1.40. The number of skin pores decreased by 24.83%, from 125.39 to 94.23. The skin pore size decreased from 0.07 to 0.02 µm^3^ (71.43% decrease). The amount of extracted sebum increased by 335% when the cleanser was used (Lee BM. et al., 2015). Compared to the control cleanser, the skin oil content was significantly decreased when the cleanser that contained HZ was used. Joanna focused on resveratrol (25), which was obtained from HZ. The study was performed in a group of 20 volunteers over a period of 6 weeks (Igielska-Kalwat et al., 2019). After 6 weeks, the measurements taken in the 4th week showed an increase in hydration by 201%, which was a much higher effectiveness than the prepared emulsion without resveratrol (25).

In addition to the utilization in humans, HZ is often used as animal feed. It was found that HZ not only did not harm mountain chickens but also enhanced the immunity and reduced the morbidity (Kuang et al., 2012). Therefore, HZ is suitable for farming chickens in the mountain and forest area. A research used HZ as an auxiliary medicine to treat rales in the lungs of cattle with good results (Zhao et al., 1993). Wu also documented the efficacy of HZ in the treatment of constipation, burns, and rheumatoid arthritis in pigs (Wu 1991).

The above results showed that HZ had a high clinical application value. It could effectively alleviate the clinical symptoms and improve the quality of life. Therefore, besides adopting a variety of methods to prevent further invasion of HZ, human beings can also expand its use and turn waste into treasure. However, what is the specific mechanism of HZ clinical efficacy? Are these satisfactory clinical traits a placebo effect? All these issues need further study.

## 7 Quality Control

As a natural herb, the chemical constituents of HZ are complex. Establishing the quality control approach is significant to guarantee its stable quality. According to 2020 Edition ChP., the content of emodin (2) and polydatin (26) in HZ must be no less than 0.60 and 0.15%. In Taiwan Herbal Pharmacopeia, emodin (2) must be no less than 0.60%, while polydatin (26) must be no less than 0.80%. Hong Kong Chinese Materia Medica (HKCMM) standards only stipulate that the total content of emodin (2) and polydatin (26) should not be less than 1.10%. As seen from the above, different countries and regions have different requirements for quality control standards.

In fact, the ingredient content of HZ produced in different countries and regions does vary. Chen reported on the quantification of resveratrol (25) and polydatin (26) in roots, stems, and leaves of HZ samples from Prince Edward Island (PEI) and nine provinces of China (Guizhou Province, Zhejiang Province, Fujian Province, Sichuan Province, Yunnan Province, Jiangsu Province, Jiangxi Province, Henan Province, Hubei Province) by ultra-performance liquid chromatography (UPLC). The results showed that the average content of polydatin (26) in PEI samples was about 11.04 mg/g, slightly higher than the average of Chinese samples (9.27 mg/g), while the average content of resveratrol (25) in Chinese samples was 4.30 mg/g, 1.6 times higher than that of the PEI samples (Chen et al., 2013). Zhang also determined the contents of polydatin (26), resveratrol (25), emodin (2), and physcion (1) in HZ produced in 13 regions of China. The results demonstrated that the contents of polydatin (26), resveratrol (25), and emodin (2) in samples from different producing areas were the highest in Xingyi, Guizhou (2.16, 0.42, 0.97%), and the lowest in Yuqing, Zunyi (0.44, 0.12, 0.61%). The content of physcion (1) was the highest in Guiyang, Guizhou (1.60%), and the lowest in Xi ‘an, Shanxi (0.36%) (Yuan et al., 2013). In addition, other scholars studied the content of polydatin (26) and resveratrol (25) in HZ from five producing areas in southwest China. The results indicated that there is no obvious regional difference in the content of polydatin (26), while the content of resveratrol (25) is quite different. The resveratrol (25) contents of the samples from the two areas in Guizhou (Zunyi and Bijie) were both higher than 0.4%, while the resveratrol (25) content of the samples from Yongchuan, Chongqing, was significantly lower than that of other production areas, only 0.09% (Zhang et al., 2012). This is because under the similar climate, soil, and other external environmental conditions, the accumulation of active ingredients in HZ is more similar. However, there are the large differences in various external environmental factors in different producing areas, so the relative ratio of the content of each component in the samples from different producing origins is different. It is suggested that the construction of standardized planting bases should be accelerated to ensure the stable and controllable quality of herbs from the source.

In addition, different growth years also have influence on the changes in the content of components in HZ. Liang found that the content of polydatin (26) was the highest at 2 years of root age (0.832%) and that of resveratrol (25) was the highest at 3 years of root age (0.201%) (Liang et al., 2011). Chen also confirmed that the levels of polydatin (26) were the highest at 2 years of root age, while the content of emodin (2) and physcion (1) reached its peak at 3 years of root age (Chen J. et al., 2020). Hence, with the development of modern separation and identify techniques, it is widely accepted that the quality of herb medicine cannot be measured only by two contents. Yuan established a quality control method for simultaneous determination of eight components in HZ by high-performance liquid chromatography-diode array detection (HPLC-DAD). This method indicates the difference of the chemical component in HZ from various habitats and can be used for quality control (Yuan et al., 2013).

## 8 Toxicology

In traditional Chinese practice, HZ is often banned for pregnant women because of the risk of miscarriage. The 2020 edition of ChP. also requires pregnant women to use it with much caution. However, at present, there is no record of poison in ancient books of HZ. According to statistics, the adverse reactions of HZ in clinical use are mainly oral preparations. The main manifestations are gastrointestinal system damage, such as diarrhea, nausea, abdominal pain, and other symptoms (Liu et al., 2018a). Free anthraquinone extracted from HZ (1.5, 2.0, 2.5, 3.0 mg/mL) downregulated the survival rate of HepaRG cells and induced the apoptosis of HepaRG cells in a dose-dependent manner, which suggested that free anthraquinones might be the important components inducing toxicity in HZ (Wang et al., 2020). However, it was reported that the oral administration of anthraquinones (9 g/kg) did not cause death in mice in a maximal tolerance dose test, and the LD50 of emodin (2) and polydatin (26) were 249.5 ± 734.3 mg/kg and 1,000 ± 757.3 mg/kg, respectively (Peng et al., 2013). Under experimental conditions, polydatin injection (0.39 mg/mL) showed no hemolysis *in vitro* or agglutination reaction. It had no systemic anaphylaxis in guinea pigs (5.6 mg/kg) or passive skin allergy in rats (5.6 mg/kg). It either had no stimulating effect in rabbit auricular vessels and muscles (5.6 mg/kg) (Xu et al., 2008). In the teratogenic sensitive period of pregnant rats, no maternal toxicity was observed when the intravenous dose of polydatin (26) was 15 mg/kg, 30 mg/kg, and 60 mg/kg (7.5, 15, and 30 times of the human clinical dose, respectively). Also, no abnormalities were observed in fetal mice (Wei et al., 2005). However, injection of polydatin (26) could dose-dependently induce peritonitis in a subacute toxicity test (Peng et al., 2013). At present, the relative systematic toxicity and safety investigation of this plant were lacking; few evaluations of target–organ toxicity or side effects had been documented. Until now, the relative systematic toxicity and safety investigation of this plant were lacking; more evaluations of target–organ toxicity or side effects needed to be documented.

## 9 Discussion and Conclusion

The present review summarized the botany, ethnopharmacology, phytochemistry, pharmacological activity, clinical use, quality control, and toxicology of HZ, which is a well-known genuine Chinese herbal medicine with the medicinal history for thousands of years. To date, 110 compounds have been found in HZ. Anthraquinones and stilbenes, the major compounds presenting in HZ, have anti-tumor, anti-oxidant, anti-pulmonary fibrosis, and anti-viral effects. Clinically, HZ is widely used in burns, various skin inflammations, gout, and other diseases. In addition, it is widely used in health products, cosmetics, and even animal husbandry feed and has no obvious toxicity. As Heinrich puts it, “controlled clinical trials or rigorous biomedical research” is needed if we want the use of medicinal plants to become a science-based medical practice (Heinrich et al., 2020). Although there are many reports, gaps still exist in the scientific studies on HZ. Therefore, we provide several topics which should have priority for further detailed investigation.

First, HZ has many synonyms, such as Yinyanglian (in Chinese), Kojo-kon (in Japanese), Itadori-kon (in Japanese), Hojang (in Korea), Japanese knotweed, bushy knotweed rhizome, giant knotweedand, and Mexican bamboo. Ma has counted that the different names of HZ amount to 170 because of the different literature studies, territories, and nationalities (Ma et al., 2006). This is very likely to lead to clinical medication confusion, with safety risks. It also makes laboratory studies of knotweed more difficult to ensure that what is being used is genuine. Therefore, the methods of literature review and field investigation should be adopted to further standardize the names of medicinal materials and strictly adhere to the correct names prescribed by pharmacopoeia in the process of application.

Second, HZ has been well documented in the China Pharmacopeia as drugs to invigorate the blood, dispel stasis, clear heat, and resolve toxicity in clinical applications. Modern pharmacological research studies have certified that HZ eliminates wind and humidity. It is effective in the treatment of gout. Moreover, other traditional uses of HZ such as dispelling stasis, stopping pains, and treating burns and scald have also been gradually substantiated by modern pharmacological studies except the application of relieving cough and reducing sputum. According to TCM theory, HZ can be used for cough due to lung heat. HZ is also often used with Scutellariae Radix (Huang Qin, HQ in Chinese), Lonicerae Japonicae Flos (Jin Yin Hua, JYH in Chinese), and Eriobotryae Folium (Pi Pa Ye, PPY in Chinese) for cough. However, at present, there is no detailed pharmacological experiment or chemical component research data to prove its effect on curing cough. Further study can delve deeper into this question.

On the other hand, many of the pharmacological effects we have discovered, such as the regulation of endocrine systems, have not been documented traditionally. The pharmacological model is too complex, and additional experiments require to be conducted. Researchers try to prove that a certain medicine (local or traditional) is effective, but the scientific method used is fundamentally flawed. For example, for many years, anti-bacterial activity has been determined by biocidal and bioinhibitory assays. If the plant extract was inactive in these assays, it was discarded and marked as having no anti-microbial activity. It has now been discovered that many botanicals may exert their anti-bacterial activity through different mechanisms of action (Heinrich et al., 2020). Therefore, this reminds us that we should not be constrained by tradition, but we should actively explore new effects and continuously expand the clinical application of HZ.

Finally, the root of HZ is used as the effective agent in TCM. However, the aerial part of this plant is commonly disposed in landfills without usage, although this part weighs no less than 50% of the total mass of the plant (Peng et al., 2013). Although little investigation has been done on it at present, important uses have been gradually found. For example, Sun found that the flowers of HZ had a very significant homicidal activity against Lucilia sericata, showing such behaviors as attracting, exciting, anesthesiaing, convulsing, and dying and the body mummified (Sun et al., 2015). It has a good prospect of developing into new green insecticides. Another example is the leaf of HZ, which could treat headache, dizziness, tinnitus, palpitation, and insomnia caused by liver yin deficiency with hyperactivity of liver yang in clinical applications (Wang et al., 2019b). Hence, it is essential to research the chemical constituents and pharmacological effects of the aerial part and find new chemical components in order to reuse the aerial part as value-added products of HZ.

In conclusion, with increasing interest of HZ in recent years, more and more phytochemical and pharmacological studies will update our knowledge of HZ. We should continue to augment the basic study and utilize the global surplus resources of HZ to develop more products such as effective drugs, health care products, cosmetics, and agricultural and animal husbandry products to benefit mankind.

## Data Availability

The original contributions presented in the study are included in the article/Supplementary Material; further inquiries can be directed to the corresponding authors.
